# Virtual Screening in the Identification of Sirtuins’ Activity Modulators

**DOI:** 10.3390/molecules27175641

**Published:** 2022-09-01

**Authors:** Elena Abbotto, Naomi Scarano, Francesco Piacente, Enrico Millo, Elena Cichero, Santina Bruzzone

**Affiliations:** 1 Department of Experimental Medicine, Section of Biochemistry, University of Genoa, Viale Benedetto XV 1, 16132 Genoa, Italy; 2 Department of Pharmacy, Section of Medicinal Chemistry, School of Medical and Pharmaceutical Sciences, University of Genoa, Viale Benedetto XV, 3, 16132 Genoa, Italy

**Keywords:** sirtuins, virtual screening, docking, rational design, activators, inhibitors, cancer, neurodegenerative disease, type 2 diabetes

## Abstract

Sirtuins are NAD^+^-dependent deac(et)ylases with different subcellular localization. The sirtuins’ family is composed of seven members, named SIRT-1 to SIRT-7. Their substrates include histones and also an increasing number of different proteins. Sirtuins regulate a wide range of different processes, ranging from transcription to metabolism to genome stability. Thus, their dysregulation has been related to the pathogenesis of different diseases. In this review, we discussed the pharmacological approaches based on sirtuins’ modulators (both inhibitors and activators) that have been attempted in in vitro and/or in in vivo experimental settings, to highlight the therapeutic potential of targeting one/more specific sirtuin isoform(s) in cancer, neurodegenerative disorders and type 2 diabetes. Extensive research has already been performed to identify SIRT-1 and -2 modulators, while compounds targeting the other sirtuins have been less studied so far. Beside sections dedicated to each sirtuin, in the present review we also included sections dedicated to pan-sirtuins’ and to parasitic sirtuins’ modulators. A special focus is dedicated to the sirtuins’ modulators identified by the use of virtual screening.

## 1. Introduction

**Figure 1:**
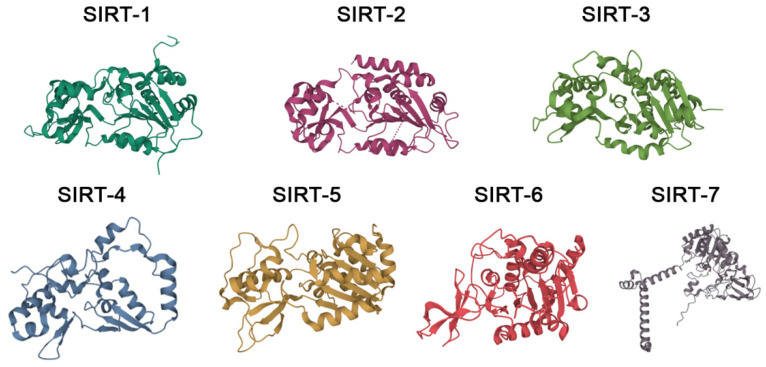
**Crystal structures of the 7 human sirtuins**. The PDB identifiers for SIRT-1, SIRT-2, SIRT-3, SIRT-5, and SIRT-6 are 4KXQ, 4Y6O, 4BN4, 6LJK, and 6HOY, respectively. SIRT-4 and SIRT-7 do not exist in a crystal structure; thus, the AlphaFold-software-predicted structures are shown with the identifiers AF-A0A347ZJG7-F1 and AF-Q9NRC8-F1, respectively.

Sirtuins (SIRTs) are a family of evolutionary, conserved enzymes that depend on nicotinamide adenine dinucleotide (NAD^+^) and are homologues of the silence information regulator 2 (Sir2) enzyme, thus also being known as Sir2-like proteins [1]. In mammals, the seven known sirtuins, named SIRT-1 to SIRT-7, are classified as class III histone deacetylases (HDACs). Their substrates, however, have been reported to be also non-histone proteins, such as cytoskeletal proteins, signaling molecules, transcription factors, chaperones, p53 and DNA repair proteins [2,3,4]. Sirtuins, moreover, often possess other enzymatic activities apart from the deacetylation one, such as deacylation and mono-ADP-ribosylation [2,3,4,5]. Structurally, the seven isoforms share a central catalytic domain of about 270 amino acids, where a Rossman fold and a smaller domain with the NAD^+^-binding module and a zinc-binding one create the enzymatic active site; the seven sirtuins then differ in the N-terminal and C-terminal domains [6]. The crystal structures of the seven sirtuins, chosen on the basis of resolution values (<2.00 Å), are represented in Figure 1.

Sirtuins are characterized by diverse subcellular localizations, unique substrate specificity and distinct enzymatic activities (Table 1) [7], and this confers to each isoform’s specific functions. Given their involvement in different biological pathways, ranging from transcription to metabolism to genome stability, their dysregulation is implicated in many diseases, such as cancer, neurodegenerative disorders, diabetes, and cardiovascular and autoimmune diseases [8,9,10,11,12]. Pharmacological modulation of their enzymatic activity becomes, therefore, a promising strategy to modify disease initiation and/or progression. Extensive research has already been conducted on SIRT-1 modulators, while compounds targeting the other sirtuins have been less studied or faced issues such as reduced isoform selectivity, poor water solubility or limited cell membrane permeability.

In the first part of the present review, we summarize the sirtuins’ modulators that have demonstrated biological effects in cellular or animal models in cancer, neurodegenerative diseases and type 2 diabetes (T2D), to demonstrate the great potential of these enzymes as therapeutic/drug targets. In the second part, a special focus will be dedicated to the sirtuins’ modulators identified by the use of virtual screenings (VS).

## 2. Sirtuins as Targets in Different Pathologies

Sirtuins play a critical role in cancer and have been reported with a dual function in tumorigenesis, behaving as oncopromoter or oncosuppressor, depending on the sirtuin and on the cancer type [28]. In addition, often one same sirtuin has controversial roles within the same cancer type, with independent studies reporting that activators/overexpression or inhibitors/knock down have both anti-cancer effects [28]. The possibility that sirtuins act as a tumor suppressor is mainly corroborated by their role in maintaining genome stability through chromatin regulation and DNA repair. On the other hand, some sirtuins were found to be overexpressed in certain tumors and were also shown to promote angiogenesis. Moreover, sirtuins affect metabolism, thus acquiring pro-tumorigenic or oncosuppressive functions. For instance, one reason to consider SIRT-6 as an oncosuppressor is represented by the shift from aerobic respiration to glycolysis observed in SIRT6-deficient cells resembling the Warburg effect, typical of cancer cells. However, the number of pathways affected by the different sirtuins is so wide that in a different cancer context the final outcome of sirtuins’s contribution can be dual. Of note, changes in sirtuins’ expression are often a consequence of time, and not a cause of different oncogenic pathways. The most studied SIRTs in cancer are SIRT-1, -2, -3 and -6, with modulators that either exhibit an anti-proliferative effect, induce apoptosis, or sensitize cancer cells to existing chemotherapeutics in vitro, or that block tumor growth in vivo.

Sirtuins are also involved in many neurodegenerative diseases, among which Alzheimer’s disease (AD), Parkinson’s disease (PD), Huntington’s disease (HD), amyotrophic lateral sclerosis (ALS) and multiple sclerosis (MS) are the most common. Neurodegenerative diseases are characterized by neuronal cell death, which leads to progressive loss of mobility, coordination, sensation and memory, and current treatments aim solely at relieving physical or mental symptoms, since no cures exist for any neurodegenerative diseases [29]. Sirtuins’ modulators proved to be effective in reducing the symptoms and in some cases also in preventing progression of neurodegeneration (AD, PD, ALS) or inflammatory relapses (MS). SIRT-1 and -2 (with some research conducted also on SIRT-3 and -6) represent the most investigated SIRTs under a pharmacological perspective. In addition, genetic studies have been also conducted on the other sirtuins, demonstrating the potential of this class of proteins as drug targets in neurodegenerative diseases [11]. All sirtuins, however, do not possess the same function in neurodegenerative diseases, with some that are downregulated and others that are upregulated in disease progression.

Regarding type 2 diabetes (T2D), some studies have been carried in the past decades to define the role of SIRTs [8], while less studies involving SIRTs modulators have been performed. In vivo experiments demonstrated that effective treatments were obtained with SIRT-1 activators, or SIRT-2 and SIRT-6 inhibitors.

The sirtuins’ pharmacological modulation that has been reported to exert therapeutic effects in vitro and in vivo in a selection of diseases, namely, cancer, neurodegenerative diseases and T2D, is summarized in Table 2.

In the following summary of sirtuins’ inhibitors and activators, those that also modulate other enzymes (e.g., kinases or others) and therefore that are not specific for the sirtuin family, have not been reported, since their beneficial effects may not be entirely attributed to the sirtuin modulation.

### 2.1. SIRT-1 Modulators as Therapeutics

SIRT-1 is the best characterized isoform of the sirtuin class of enzymes: it is located in the nucleus and it regulates gene stability, stress response and apoptosis [30,31,32]. Its modulators have been extensively investigated in the past decades [33]; a particular mention should be conducted of the inhibitor EX-527, which is selective for SIRT-1 over the other sirtuins, and furthermore has been also used in clinical trials as treatment for HD. Other inhibitors did not prove to be suitable for clinical studies until now, even though many were explored in in vitro and in vivo settings. Resveratrol and the compounds developed by the company Sirtris Pharmaceuticals, SRT1720, SRT2183, SRT1460, have been extensively used in disease models as SIRT-1 activators. However, Pacholec and colleagues demonstrated that they are not direct activators of SIRT-1 [34], and therefore even though they exhibited therapeutic effects in many diseases and SIRT-1 was modulated [35,36,37,38,39,40,41], they are excluded from the below summary of SIRT-1 activators.

#### 2.1.1. SIRT-1 Modulators in Cancer

In cancer, SIRT-1 has a controversial role, being reported with oncopromoter and oncosuppressor functions. This was also confirmed by several studies with inhibitors that were shown as promising anti-cancer agents, and with activators that had positive biological effects as well, in some other cancer types.

SIRT-1 inhibition has been shown to exert a great therapeutic effect in several types of cancers. In two independent studies, **Selisistat** (called also **EX-527** or **SEN0014196** and discovered by a high-throughput screen in 2005) [42] and compound **JGB1741** (developed starting from the structure of Sirtinol and modifying its structure on the basis of its probable interaction with the protein SIRT-1) [43] induced apoptosis in leukaemia cells. The induction of apoptosis was also observed upon the treatment of breast cancer and HCC cells with compound **JGB1741** [43]. **EX-527**, which has a selectivity 200 times higher for SIRT-1 with respect to the other sirtuins, on the other hand, has been investigated in several other cancer types: in glioma [44], bladder [45], and endometrial [46] cancers, it exhibited anti-proliferative properties; in pancreatic cancer, it blocked cell proliferation and sensitized its cells to gemcitabine [47]; in endometrial [46] carcinoma, it reduced cancer cell proliferation and tumor growth in mouse studies. In lung cancer, inhibition was demonstrated to have several anti-cancer effects, with research showing that **EX-527** increased sensitivity to MK-1775, as well as reduced tumor growth in an in vivo mouse model [48] and increased sensitivity to cisplatin in vitro [49]. Breast cancer has been investigated with SIRT-1 inhibitors: two studies with EX-527 showed that it causes cell cycle arrest [50] and suppresses the resistance to Hsp90 inhibitors in cancer cells [51]. Other works indicated that compound **4d** [52], which was discovered by VS as well (see Section 3.2), compound **3a** and its family of Aurone derivative, synthetized starting from a thiobarbiturate structure (a promising inhibitor of sirtuins), [53] and compounds **27** and **30**, obtained from a synthesized library of pseudopeptides, [54] inhibited cell proliferation; in addition, inhibitor **Amuresin G**, obtained from a plant extract of *V. amurensis*, increased cellular uptake of doxorubicin in doxorubicin-resistant breast cancer cells and restored the responsiveness of MCF-7/ADR cells to doxorubicin in vitro and in vivo. [55]

SIRT-1 inhibitors have been investigated in prostate cancer as well: **EX-527** increased the sensitivity of cancer cells to vesicular stomatitis virus oncolysis [56], while compound **17** [57] (discovered by VS, see Section 3.1) exhibited anti-proliferative effects in vitro. 

In colon and NSCL cancer, inhibitor **Inauhzin** (found by a computational structure-based screening) decreased cell proliferation, induced senescence and apoptosis and inhibited tumor growth in mouse models. [58] One more compound to mention is inhibitor **S1th**, which was discovered by VS (see Section 3.2) and was reported to possess anti-proliferative proprieties in cervical cancer cells [59].

The mechanism of action of all these SIRT-1 inhibitors in tumors is the induction of cell cycle arrest and tumor cell apoptosis via the p53 pathway, increasing Bax/Bcl-2 ratio and PARP cleavage.

SIRT-1 pharmacological activation was also demonstrated to have anti-cancer effects. In particular, in neuroblastoma **Chikusetsu saponin V** (extracted from *Panax japonicus*) reduced H_2_O_2_-induced oxidative stress in cell studies by increasing the activity of SOD and increasing the levels of GSH [60].

#### 2.1.2. SIRT-1 Modulators in Neurodegenerative Disorders

Neurodegenerative diseases treatment may benefit from SIRT-1 inhibitors as well as activators.

In particular, inhibitors **EX-527** [61] and **Baicalin** (isolated from the roots of the *Radix Scutellariae* plant) [62] were demonstrated to have a neuroprotective effect in mice, by reducing acute stress response and by negatively regulating the expression of IL-6, TNF-α, and Il-1β in the hippocampus and hypothalamus through the regulation of the SIRT-1-NF-kB pathway.

On the other hand, SIRT-1 activators also exhibited protection from neuroinflammation. Specifically, **Salidroside** (extracted from *Rhodiola rosea L.*) reduced inflammation-induced cognitive defects in mice through the Nrf-2/HO-1/NF-κB pathway [63], and 17β-estradiol blocked neuroinflammation and neuronal apoptosis, halting cognitive dysfunction and memory impairment in a male aging mouse model [64]. 

In **AD,** pieces of evidence revealed that SIRT-1 activation may be protective, while pharmacological inhibition aggravated tau accumulation [65] and abolished the resveratrol-mediated attenuation of autophagy in an AD cell model [66]. Other examples of activators/molecules causing SIRT-1 overexpression with a biological effect in AD are ***Syzygium aromaticum* extract** (obtained by the ethanol extraction of a pool of molecules from *Syzygium aromaticum*), which instead maintains oxidative balance in vitro and in AD patients’ serum by increasing the expression and activity of SOD and GSH [67], and **dihydromyricetin** (extracted from *Ampelopsis grossedentata*), which inhibits neuronal cell apoptosis, ameliorating cognitive dysfunction in the AD rat model by activating the AMPK/SIRT-1/PGC-1α pathway [65,68]. Other pieces of evidence include the **SLAB51** probiotic formulation, which reduces oxidative species levels in AD mouse, thus having a neuroprotective effect by activating SIRT-1 and increasing the oxidative stress defences [69] and **Cilostazol**, which suppresses β-amyloid production in vitro by stimulating the expression of α-secretase via the ADAM10/SIRT-1 pathway activation [70], thus improving or protecting cognitive function, through increased glucose metabolism, in AD patients with white matter lesions [71]. 

In **HD**, conversely, SIRT-1 inhibition seems to be required in order to gain neuroprotection. Indeed, the inhibitor **EX-527** displayed neuroprotection in Drosophila and in mouse models of HD, by improving motor function through inactivation of the FOXO3A and CREB pathways and the subsequent decrease in the expression of exon 1 of the Htt protein [72], and has been further studied in a few clinical trials (Phase I and II) [73,74]. Specifically, in two studies in healthy volunteers and HD patients, EX-527 was found to be well tolerated with no adverse effects, even though, unfortunately, the circulating levels of soluble huntingtin were not affected. Nevertheless, two other studies in the HD mouse model showed that increasing NAD^+^ availability and causing SIRT-1 activation with **β-lapachone** (a natural o-naphthoquinone compound, and a substrate of NADH:quinone oxidoreductase, NQO1) results in neuroprotection by activating autophagy of Htt exon1-expressing cells [75,76], revealing that further investigations involving sirtuin modulators and HD are still needed.

In **PD,** SIRT-1 proved to be neuroprotective when activated, as indicated by some pharmacological studies, including the use of the activators **Echinacoside** [72,73] derived from the fleshy stem of distance, a widely used Chinese herb [72,77], and **Embelin**, a natural product with structural resemblance to ubiquinone, exhibiting mitochondrial uncoupling and antioxidant effects [73,78], which are neuroprotective in an induced neurotoxic PD mouse model. **Echinacoside** mediates the autophagic degradation of α-synuclein by the increased expression of FoxO1 via SIRT-1 activation [77]. The mitochondrial uncoupling effect of **Embelin** increases NAD^+^/NADH levels, followed by enhanced SIRT-1, PGC1α and mitochondrial biogenesis conferring to **Embelin** neuroprotective properties [78].

Another neurodegenerative disease to be noted is **ALS**, for which the inhibitor **EX-527** has shown a neuroprotective effect in an in vitro study, by increasing neuron viability [74], although with an action independent from SIRT-1 inhibition [74,79]. No studies have been performed with activators or with other inhibitors; therefore, there is potential for further investigation in this field.

**Multiple Sclerosis (MS)** is the last neurodegenerative disease covered in this review and a few studies were reported showing the anti-inflammation proprieties of inhibitor **EX-527**. In one study, EX-527 expanded the endogenous pool of neuronal progenitor cells without affecting their differentiation perhaps by increasing PDGFRα expression and the activity of p38 MAPK and AKT pathways [80]; in an additional study, it was able to suppress the cell differentiation of Th17 by inhibiting RORγt transcriptional activity, decreasing Th17 cell generation and function. Th17 are cells associated with multiple autoimmune diseases, to delay disease onset and reduce lymphocytic infiltration and demyelination in an in vivo mouse model [81].

#### 2.1.3. SIRT-1 Modulators in T2D

SIRT-1 regulates glucose/lipid metabolism, since it deacetylates several proteins of the insulin signaling pathway, and its overexpression improves the sensitivity to insulin. Some SIRT-1 activators, such as **JHJ1**, **JHJ2**, and **JHJ3** (derived from the structure of OAP) [82], have been reported to modulate the plasma lipid metabolism and blood glucose in high-fat fed mice by upregulating FoxO1, PPARγ, and PGC-1α genes [83], while another activator, named **E6155** (identified by a high-throughput screening using the purified recombinant human SIRT-1 and HTRF SIRT-1 assay) by Liu and colleagues, improved blood glucose tolerance and insulin resistance in diabetic mice by activating LKB1/AMPK and IRS1/AKT pathways [84].

### 2.2. SIRT-2 Modulators as Therapeutics

SIRT-2 is localized in the cytoplasm, although it can shuttle to the nucleus to participate to several physiological and pathological functions [85]. It plays a role in several processes, including cell cycle regulation and metabolism [86,87]. SIRT-2 activators have never been discovered, while many inhibitors have been identified in the past years, with the most studied one being **AGK2**. Although this compound is the most selective inhibitor for SIRT-2, its poor water solubility creates the need to seek still for new SIRT-2 inhibitors. Since many SIRT-2 inhibitors have been studied also in vitro and in vivo, a selection of them is reported in the next paragraphs.

#### 2.2.1. SIRT-2 Modulators in Cancer

In many types of cancer, SIRT-2 acts as a tumor promotor and therefore its inhibition may be beneficial. Often, SIRT-2 inhibitors act as anti-proliferative agents or have a cytotoxic effect, since this sirtuin is involved in cell cycle regulation, in particular in the G(2)/M transition [87]. Nevertheless, in some other cancers, it was shown that SIRT-2 possesses tumor suppressor functions, which, however, were not further investigated pharmacologically, since no SIRT-2 activators are currently available. 

In glioma, SIRT-2 inhibition has shown relevant therapeutic effects: **AGK2** [88], as well as **NH4-13** [89], reduced cancer cell proliferation, and **AGK2**, in addition, displayed anti-proliferative activity in cancer stem cells [90]; moreover, another SIRT-2 inhibitor, called **AK7**, was shown to reduce tumor growth in an in vivo mouse model [88]. In particular, Funato and colleagues showed that the SIRT-2-mediated inactivation of tumor suppressor p73 is crucial in the proliferation and tumorigenicity of glioblastoma cells, thus making SIRT-2 inhibition by **AGK2** or **AK7** a valid anti-cancer treatment [88].

Compound **AC-93253** and **Salermide** (chosen at a concentration at which SIRT-2, but not SIRT-1, is inhibited) blocked cell proliferation and downregulated the c-Myc and N-Myc oncoproteins in neuroblastoma and in pancreatic cancer [91]. In addition, in this last type of cancer, other inhibitors were reported to possess an anti-proliferative activity, such as **NPD11033** [92], **AF8** [93] and **NH4-13** [89]. One more inhibitor, namely, compound **AC-93253**, then displayed a cytotoxic effect in pancreatic cancer cells, by triggering apoptosis [94]

Many SIRT-2 inhibitors displayed their anti-cancer activity by blocking cell proliferation. Examples include **SirReal2** [95] and **TM** [96] in colorectal cancer; compounds **2** and **3** in adenocarcinoma (which were discovered by VS, and mentioned in Section 3.2) [97], **SirReal2** [95], 4-chromanone compounds **6f** and **12a** [98], compound **24a** [99] and **NH4-13** [89] in lung cancer; compound **35** in NSCL cancer [100]; **NCO-90** and **NCO-141** [101], **TM** [96], compounds **35** and **39** [100] in leukaemia; **SirReal2** [95], compounds **35** and **39** [100], the Cambinol analogues compound **24** [102] and compounds **55** and **56** [103] in lymphoma; **AF8** [93], **NH4-13** [89], **SirReal2** [95], compound **35** [100], compound **6f** and **12a** [98] **RK-91230156** [104], Splitomicin derivatives compounds **5c**, **8c** and **(R)-8c** (discovered by VS, see Section 3.2) [105], **γ-mangostin** [106], **Tenovin-D3** [107], **RK-9123016** [104], compounds **2** and **3** [97] in breast cancer; **SirReal2** [95], **compounds 2** and **3** [97] and **NH4-13** [89] in cervical cancer. 

The anti-proliferative effects of these compounds were described in several works together with other molecular mechanisms affected by SIRT-2 inhibition. For instance, compound **TM** was reported to promote ubiquitination and degradation of c-Myc oncoprotein in different cancer cell lines [96], while compounds **35** and **39** induced apoptosis in leukaemia and breast cancer cells [100]. Moreover, cell viability of cancer cells was reduced because inhibitor **RK-9123016** was accompanied by a decrease in c-Myc expression [104]; in another study, SIRT-2 inhibition by **Tenovin-D3** promoted expression of the cell-cycle regulator and p53 target p21WAF1/CIP1 (CDKN1A) in a p53-independent manner, along with a reduction in cell proliferation [107]. **NCO-90/141** simultaneously caused apoptosis and autophagy in leukemic cell lines, by inducing apoptosis via caspase activation and mitochondrial superoxide generation, and by increasing the LC3-II level together with autophagosome accumulation, indicating autophagic cell death [101]. In a similar way, the induction of apoptosis was observed along with cell growth arrest when treating lymphoma cells with compounds **55** and **56** [103].

The therapeutic effect of some other SIRT-2 inhibitors relies on the combination of the cytotoxicity of the compounds and their inhibitory activity, having been observed that the compound toxicity was crucial in obtaining anti-cancer effects; this is the mechanism suggested for **AC-93253** in prostate, pancreatic and lung cancer [94]. 

In addition, some inhibitors have been further studied in in vivo mouse models and blocked tumor growth. These studies were performed in colorectal cancer with compounds **AGK2** [108], **AF8** [93] and **NH4-13** [89]; in gastric cancer using **SirReal2** [95]; in breast cancer with compound **TM** [96]. 

From the point of view of the isoform-selectivity of the compounds, the most selective SIRT-2 inhibitors listed above are **AGK2**, **AK7**, **NH4-13, NPD11033**, **AF8**, **AC-93253**, **SirReal2**, **TM** and **RK-91230156.** In particular, **AGK2**, which was identified from a screening of 200 compounds [109], inhibits SIRT-2 with an IC_50_ of 3.5 μM, and inhibits SIRT-1 and -3 with IC_50_ of 30 and 91 μM, respectively [110], while **NH4-13** inhibits SIRT-2 with an IC_50_ of 0.087 μM, and its IC_50_ for SIRT-1, -3, -5 and -6 is reported to be higher than 50 μM [89]. Inhibitor **AK7** was identified after making a substructure search of sulfobenzoic acid derivatives that were predicted to be brain permeable, since the authors were targeting HD and had already identified inhibitors with this core structure. **AK7** was reported to be brain permeable in vivo, with an IC_50_ of 15.5 μM, and to neither modulate SIRT-1 or -3 up to 20-50 μM [111]. **NPD11033**, which was discovered by a high-throughput screen method has an SIRT-2 IC_50_ of 0.46 μM, while its IC_50_ for SIRT-1 and -3 is higher than 100 μM [92]. One more inhibitor is **AF8**, which is characterized by an IC_50_ of 0.06 μM for SIRT-2, and of 11 μM or above 50 μM for SIRT-1 and -3, respectively [93]. The inhibitory ability of **AC-93253** was tested in vitro and not in enzymatic assays, and it was reported that its IC_50_ was 6 μM, and that it was 7.5- and 4-fold more potent in inhibiting SIRT-2 than the isoforms SIRT-1 and -3 [94]. **SirReal2** was identified through an in vitro compound screening of an in-house developed library [112], and its IC_50_ for SIRT-2 is 0.23 μM, while it is above 50 μM for SIRT-1 and -3 [95]. **TM** is another SIRT-2 selective inhibitor, with an IC_50_ values of 0.4 μM for SIRT-2, and it is at least 650-fold more selective for SIRT-2 compared to SIRT-1 and -3 [95]. **RK-91230156**, finally, has an IC_50_ for SIRT-2 of 0.18 μM, while it is above 100 μM for SIR-1 and -3 [104].

Less selective inhibitors, but still to be mentioned, are the following compounds. Compounds **6f** and **12a**, which were discovered after optimizing the hydrophilicity of an inhibitor previously developed by the group, have IC_50_ of 3.7 and 12.2 μM, respectively, while their IC_50_ for SIRT-1 and -3 are above 200 μM [98]. **Tenovin-6**, instead, was discovered by screening, in a cell-based assay, 30,000 drug-like small molecules present in the Chembridge DIVERSet, making a selection of those that had been reported to activate p53 [113], and exhibits IC_50_ for SIRT-2, -1 and -3, respectively, of 9, 26 and above 50 μM [95]. Moreover, Yang and colleagues demonstrated that compound **24a**, which was identified with X-ray crystal structure-guided structure–activity relationship (SAR) studies, has an IC_50_ value of 0.815 μM; the modulation of other sirtuins, however, was not investigated [99]. Moreover, compounds **35** and **39** were discovered after SAR studies performed on an existing SIRT-2 inhibitor, and they proved to be quite effective in inhibiting SIRT-2 (IC_50_ 10.4 and 1.5 μM, respectively) and with a good selectivity for this isoform (further studies on compound **39** with SIRT-1, -3, -5 revealed no non-specific effects up to 100 μM) [100]. Splitomicin derivatives, compounds **5c**, **8c** and **(R)-8c**, were discovered by VS (see Section 3.2) and have been reported with IC_50_ ranging from 1.0 to 1.5 μM (the modulation of other sirtuins was not investigated) [105]. **γ-mangostin** is one other SIRT-2 inhibitor to mention, since it displays ≥6-fold selectivity against SIRT-2 (SIRT-2 IC_50_ = 3.8 µM; SIRT-1 and -3 IC_50_ in the 22–26 µM range); this xanthone extracted from the tropical plant *Garcinia mangostana* was investigated after it was reported that the α-isoform of mangostin was able to modulate sirtuins [106].

#### 2.2.2. SIRT-2 Modulators in Neurodegenerative Disorders

Among different tissues, SIRT-2 expression is most abundant in the brain, at the neurons and oligodendrocytes levels; thus, the role of SIRT-2 in neurodegenerative disorders has been extensively investigated in the past years [77,114]. SIRT-2 inhibitors have been shown to have a neuroprotective effect in the main neurodegenerative disorders. In particular, Wang and colleagues showed that inhibitor **AGK2**, which was proved to be brain permeable, was able to significantly decrease LPS-induced neuroinflammation markers in cell and mice models [115]. Furthermore, another inhibitor, namely, **AK1**, provided some neuroprotection in the hippocampus, in which inflammation is associated both to Alzheimer’s disease and tau-associated frontotemporal dementia [116].

It is worth noting that inhibitor **AK1** was discovered along with the previously described **AGK2** and it is selective for SIRT-2; however, **AK1** is slightly less potent than **AGK2** (12.5 μM vs 3.5 μM) [109].

In **AD**, several SIRT-2 inhibitors have been shown to be promising therapeutics, both in cell and mouse models. In cellular studies, SIRT-2 inhibitors displayed different mechanisms in neuroprotection: in one study, **γ-mangostin** induced neurite outgrowth in an AD cellular model, [81,106] in another one, **AGK2** reduced the reactive gliosis (which is considered one of the hallmarks of AD), [40] while in one other study, **AK1** modulated mitochondrial dysfunction in AD cells, by recovering microtubule stabilization, and by eliminating toxic Aβ, thus improving cell survival [84,117]. Collecting instead the evidence of the in vivo research conducted, one study reported that compound **33i** was effective in hampering age-related cognitive decline in a senescence-accelerated mouse model, by preventing neuroinflammation, having observed reduced levels of GFAP, IL-1β, Il-6, and Tnf-α [118]. Another work demonstrated that inhibitor **AK7** decreased the BACE1 and Aβ production in an AD mouse model, improving the cognitive functional defects of the mice; [86,119] one more study reported that **AK7**, via SIRT-2 inhibition, decreased the phosphorylated tau levels, a characteristic linked to tau pathology, resulting in increased Tau/tubulin and α-synuclein/tubulin binding, thus reducing tau and α-synuclein aggregation and neurotoxicity [120]. 

In addition, Biella and colleagues observed a reduction in the Aβ production in neuroglioma cells and a modification in the Amyloid Precursor Protein proteolytic processing, when SIRT-2 was inhibited with **AGK2** or **AK7** in two AD transgenic mouse models. While the amount of soluble Aβ decreased, the one of soluble α-amyloid protein increased, thus improving the cognitive performance of mice [88,121].

**HD** is another neurodegenerative disorder in which SIRT-2 inhibition is to seek. Some pieces of evidence are here described. Quinti and colleagues reported the neuroprotective effects of inhibitor **MIND4** in ex vivo brain slices and in Drosophila models of HD, where the induction of cryoprotective NRF2 responses in neuronal and non-neuronal cells was seen, accompanied by reduced production of ROS and nitrogen intermediates [89,122]. Likewise, inhibitors **AK1** and **AGK2** also decreased Huntingtin accumulation and increased neuronal viability in Drosophila [90,123]. Moreover, two more studies in a genetic mouse model of HD demonstrated that inhibition with **AK7** disrupted disease progression, by improving motor function, extending survival, reducing brain atrophy, and reducing aggregated mutant Huntingtin in the mice [88,91,111,124].

The inhibitor **MIND4** was identified using an iterative structure–activity drug discovery approach, starting from a known SIRT-2 inhibitor and making chemical modifications with the aim of improving potency and selectivity [122].

In **PD**, several SIRT-2 inhibitors were proved to have neuroprotective effects. In several independent studies, **AK1**, **AGK2** and compounds **86** and **102** protected dopaminergic neurons in vitro and in a Drosophila PD model, rescuing cells from α-synuclein aggregation-induced toxicity [89,92,93,109,125,126]. Moreover, **AK7** and **AGK2** were further demonstrated to prevent dopaminergic neuronal cell death, to reduce activation of microglia and to attenuate behavioural abnormality in aged mice and rats [94,95,96,127,128,129]. Some other pieces of evidence regarding SIRT-2 inhibition in PD is given by two more studies: Di Fruscia showed that compound **10** prevented neuronal cell death, triggered by lactacystin in an in vitro model of PD, [95,130] while **AK7** was neuroprotective in vivo, by down-regulating the RNAs responsible for sterol biosynthesis [98,131].

#### 2.2.3. SIRT-2 Modulators in T2D

The function of SIRT-2 in insulin signalling is still controversial, with some authors reporting that its overexpression improves insulin sensitivity, and others demonstrating the opposite [99,132]. In fact, depending on the tissue type, SIRT-2 may exert even the opposite effect in response to insulin. For example, the SIRT-2 inhibitor **AGK2**, reproducing the effects obtained by SIRT-2 downregulation, improves insulin sensitivity in C2C12 cells (skeletal muscle cells) [89,133]. Further studies are needed with SIRT-2 activators and in different tissues in order to reach a conclusion regarding the possibility of targeting SIRT-2 in T2D.

### 2.3. SIRT-3 Modulators as Therapeutics

SIRT-3 is located in the mitochondria and has been shown to act on several metabolic and respiratory enzymes regulating their functions [134]. In addition, SIRT-3 can regulate the production and clearance of ROS by deacetylating numerous mitochondrial enzymes [135,136,137].

#### 2.3.1. SIRT-3 Modulators in Cancer

The role of SIRT-3 in cancer is controversial and there are several reports describing its function as an oncogene as well as an oncosuppressor [138]. Considering its oncogenic role, different inhibitors of the SIRT-3 deacetylase activity were developed and proved to determine cell death by increasing ROS production. This mechanism of action was reported for the compound **LC-0296** in head and neck squamous cell carcinoma (HNSCC), and for **3-TYP** in acute myeloid leukemia (AML) [139,140]. In addition, LC-0296 works synergistically to increase the sensitivity of HNSCC cells to radiation and cisplatin treatment [139]. The selectivity of LC-0296 for SIRT-3 is approximately ~20- and 10-fold greater compared to SIRT-1 and SIRT-2 (IC50 3.6, 67 and 33 μM for SIRT-3, SIRT-1 and SIRT-2, respectively) [139]. The IC50s of 3-TYP were of 16, 88 and 92 nM for SIRT-3, SIRT-1 and SIRT-2, respectively [140].

Meng Li and colleagues reported that, in lymphoma cells, compound **YC8-02** inhibits SIRT-3 deacetylase activity, bringing about an increase in mitochondrial proteins acetylation and subsequently cell death by autophagy [141]. YC8-02 was developed starting from a series of small molecules including an SIRT-2 selective thiomyristoyl lysine compound called TM and Biotin-TM3 (selective to SIRT-1 and SIRT-2). A compound called JH-T4 was developed, which could inhibit SIRT-1, -2 and -3, as measured in in vitro biochemical enzymatic assays, and increase its penetration into mitochondria, JH-T4 was modified by replacing the benzyl carbamoyl group with a triphenylphosphonium mitochondrial targeting moiety, thus producing YC8-02 [141]. This compound was more effective in inhibiting SIRT-3, although the selectivity over SIRT-1 and SIRT-3 was not obtained [141].

On the other hand, as mentioned, there are several pieces of evidence where the activation of SIRT-3 can represent a promising strategy to treat some types of tumors. The mechanism of action of SIRT-3 as a tumor suppressor seems to be different depending on the tumor type. In lung cancer, Wang and colleagues showed that the activation of SIRT-3 by Adjudin inhibits cellular growth and metastasis by regulating the SIRT-3-mediated FOXO3a axis [142]. Zhang and colleagues performed a structure-guided design of SIRT-3 activators and tested their activity on breast cancer. They found that **Compound 33c** (identified via hit-to-lead optimization starting from VS campaign, see Section 3.3) activates SIRT-3, determines breast cancer cell death by autophagy and reduces cell migration by decreasing ROS production hampering MMP activation [143]. In addition, Compound **33c** could not activate SIRT-1, SIRT-2, and SIRT-5 in an enzymatic reaction assay in vitro, thus representing a selective SIRT-3 activator [143].

In conclusion, SIRT-3, as well as other sirtuins, seems to represent a target to be either activated or inhibited in a cancer-specific manner. Thus, the research of new potent SIRT-3 modulators should proceed to look for both activators and inhibitors.

#### 2.3.2. SIRT-3 Modulators in Neurodegenerative Disorders

Regarding the role of SIRT-3 in neurodegenerative disorders, there are no reports about the use of specific modulators, but there are pieces of evidence that increasing the expression/activity of SIRT-3 could be a promising approach in a number of diseases. For example, Gastrodin can increase the levels of SIRT-3 in activated microglia causing a decrease in ROS and exhibiting a protective role [144]. In addition, Park and colleagues showed that the activation of SIRT-3 by the AMPK/CREB-PGC-1α signalling results in reduced αsyn oligomers in PD, suggesting that the use of SIRT-3 activators may represent a potential therapy to restore mitochondrial deficits and decrease αsyn-induced pathophysiology [145]. Another evidence supporting the idea that SIRT-3 activation could be a good approach in neurodegenerative disorders is that **Honokiol**, a not specific SIRT-3 activator, decreases ROS and lipid peroxidation, enhances antioxidant activities, and mitochondrial function, thereby reducing β-amyloid and sAPPβ production in an AD model. [146] Honokiol did not affect cell viability at concentrations up to 10 μM [146].

#### 2.3.3. SIRT-3 Modulators in T2D

Lee and colleagues showed that the not specific SIRT-3 activator **Honokiol** improves insulin resistance in adipocytes, by promoting insulin receptor beta (IRβ) and PI3K/AKT/mTOR pathways, resulting in an increase in phosphorylation of the forkhead family FoxO1/FoxO3a/FoxO4 and glycogen synthase kinase-3 (GSK-3β). Conversely, the SIRT-3 inhibitor 3-TYP decreases insulin resistance [147]. These data suggest that the activation of SIRT-3 with more specific activators could represent a promising approach to treat T2D.

### 2.4. SIRT-4 Modulators as Therapeutics

SIRT-4 is located in the mitochondrial matrix, where it controls several pathways by modifying the activation status of different proteins [148]. To the best of our knowledge, no specific SIRT-4 modulators are available yet. There are instead several reports suggesting the suitable therapeutical approach, targeting SIRT-4 to be adopted in different diseases.

#### 2.4.1. SIRT-4 Modulators in Cancer

Moreover, for SIRT-4, the rule that its overexpression or downregulation is cancer-specific is valid. In head and neck carcinoma, it was reported that SIRT-4 expression is induced during different types of stress, and this helps tumor cell survival [149]. In ovarian cancer, the expression of SIRT-4 correlates with a poor prognosis [150]. On the contrary, in breast cancer, neuroblastoma, liver cancer and pancreatic cancer, SIRT-4 is downregulated [151,152,153,154], suggesting an oncosuppressive role of this sirtuin.

#### 2.4.2. SIRT-4 Modulators in Neurodegenerative Disorders

Regarding the role of SIRT-4 in neurodegenerative disorders, SIRT-4 was suggested to possess potential neuroprotective roles against excitotoxic insults by facilitating glutamate uptake [155]. In a **Huntington’s disease (HD)** mouse model, SIRT-4 expression varies in different brain regions: it was increased in the striatum, decreased in the cortex, but remained unaltered in the cerebellum [156]. Thus, the correlation between SIRT-4 and neurodegeneration is still vague and it is not possible to take a stand regarding the meaning of an SIRT-4-directed pharmacological strategy in neurodegenerative disorders.

#### 2.4.3. SIRT-4 Modulators in T2D

SIRT-4 was reported to inhibit insulin secretion [157,158,159]. The molecular mechanism relies on SIRT-4 transferring an ADP-ribosyl group to glutamate dehydrogenase (GDH), thereby decreasing its enzymatic activity. In turn, GDH inhibition impairs ATP generation and insulin secretion. Thus, the pharmacologic inhibition of SIRT-4 could be proposed as a therapy for T2D.

### 2.5. SIRT-5 Modulators as Therapeutics

SIRT-5 is located in the mitochondria and controls several physiological pathways, including the promotion of ammonia detoxification, fatty acid β-oxidation and ketone body production and the regulation of energy production [160]. Several compounds were synthesized and tested as SIRT-5 modulators, but only a few of them are specific and have already been tested on diseases.

#### 2.5.1. SIRT-5 Modulators in Cancer

As for the other sirtuins, SIRT-5 also has a controversial role in cancer, being proposed as an oncogene in some cancers and as an oncosuppressor in others [160].

The most promising and selective SIRT-5 inhibitor is **DK1-04e**, which is able to suppress mammary tumor growth in vitro and in vivo [161]. IDH2, an NADPH-generating enzyme, was identified as an SIRT-5 substrate in mammary tumors: SIRT-5 was demonstrated to desuccinylate and activate IDH2 [161]. DK1-04e was obtained through chemical modifications of a thiosuccinyllysine peptide, known to inhibit SIRT-5; DK1-04e has an IC_50_ of 0.34 μM against SIRT-5, and proved to be very selective, since it showed no inhibition of SIRT-1–3 and -6 deacylation activity at 83.3 μM [161]. In addition, Yan and colleagues showed that the SIRT-5 selective inhibitor **NRD167** can inhibit AML cell proliferation, both in vitro and in vivo [162]. Cell apoptosis induced by SIRT-5 inhibition is preceded by reductions in oxidative phosphorylation and glutamine utilization, and an increase in mitochondrial superoxide [162]. NRD167 was synthesized starting from a parent known SIRT-5 inhibitor [162].

On the contrary, in pancreatic ductal adenocarcinoma (PDAC), the SIRT-5 activator **MC3138**, by promoting GOT1 deacetylation and inhibition, showed a reduction in cell viability in vitro and a reduction in tumor growth in vivo in combination with gemcitabine [163]. C3138 exhibited a selective activation of SIRT-5: it activated SIRT5 at 10 μM concentration, whereas it did not affect SIRT-1 or SIRT-3 activity at 100 μM concentration [163].

#### 2.5.2. SIRT-5 Modulators in Neurodegenerative Disorders

No reports are available on the pharmacological modulation of SIRT-5 in neurodegenerative disorders. However, the data obtained by Liu and colleagues suggest that the pharmacological activation of SIRT-5 could represent a good approach to reduce oxidative stress in PD [164]. Similarly, another study suggests that the pharmacological activation of SIRT-5 could ameliorate the progression of AD, since SIRT-5 has a role in promoting autophagy [165].

#### 2.5.3. SIRT-5 Modulators in T2D

Regarding the role of SIRT-5 in T2D, Wang and colleagues showed that SIRT-5 deficiency in brown adipose tissue (BAT) is followed by hyper-succinylation on GDH, SDHA, and UCP1, reducing their activity [166] and causing glucose intolerance. Thus, SIRT-5 activators may be a promising approach to treat T2D via BAT activation. On the contrary, Ma and colleagues showed that SIRT-5 downregulation promotes insulin secretion from β-cells, suggesting inhibition of SIRT5 as a therapeutical approach for T2D [167].

### 2.6. SIRT-6 Modulators as Therapeutics

SIRT-6 is a nuclear sirtuin and its role in the regulation of different processes is being increasingly recognized, covering many different functions, including energy metabolism derived both from glucose and lipids, DNA repair, aging, inflammation and immunity. Both activators and inhibitors of SIRT-6′s activity have been identified and evaluated, in in vitro and in vivo experimental settings.

#### 2.6.1. SIRT-6 Modulators in Cancer

Depending on the different cancer type, or even on the different cancer stage, SIRT-6 has been reported to act as an oncopromoter or oncosuppressor. These studies have been performed by overexpressing or silencing SIRT-6 expression and, depending on the obtained results, inhibitors or activators have been evoked as promising strategies.

Compound **UBCS039** is an SIRT-6 activator [168], showing no statistically significant effects on basal SIRT-1, -2, and -3 deacetylation activities and a ≈2-fold increase in SIRT-5 desuccinylation activity when added at 100 μM concentration [168]. UBCS039 [168] induced cell death and reduced cell proliferation in cancer cell lines of different origin, including non-small cell lung, colon and epithelial cervix carcinoma, and fibrosarcoma, clearly demonstrating that pharmacological SIRT-6 activation triggers an autophagy-related cell death [169]. At the molecular level, SIRT-6-mediated autophagy was triggered by an increase in ROS levels, which, in turn, resulted in the activation of the AMPK-ULK1-mTOR signaling pathway [169].

**MDL-800**, another SIRT-6 activator (developed as hit-to-lead optimization process from compounds **AN-988/40889624** and **AH-487/41802661**, discovered by structure-based studies, see Section 3.6), reduced proliferation in vitro, without inducing cell death, and suppressed tumor growth in vivo in a mouse model of hepatocarcinoma [170]. In addition, **MDL-800** reduced proliferation in non-small cell Lung cancer cells, and it was suggested that SIRT-6 activation may be promising as a therapeutic approach alone or in combination with epidermal growth factor receptor tyrosine kinase inhibitors [171]. MDL-800 potently activated SIRT-6 at ~10 µM but showed no activity toward SIRT-1, SIRT-3, SIRT-4, and HDAC1-11 at concentrations up to 50 or 100 µM [170].

**MDL-811**, derived from structural optimization of **MDL-800**, proved to represent a possible strategy against colorectal cancer, by tests conducted on the HCT116 cell line, patient-derived xenografts as well as on a spontaneous model of this cancer [172]. Mechanistically, Cytochrome P450 family 24 subfamily A member 1 was identified as a new downstream target gene of SIRT-6 in colorectal cancer [172]. As MDL-800, MDL-811 also potently enhanced the deacetylase activity of SIRT-6 but showed little effect on other histone deacetylase enzymes at concentrations up to 100 μM [172].

**Compound 12q**, designed as optimized analogue of the prototype **Hit20**, which was identified via structure-based studies (see Section 3.6), significantly inhibited the proliferation and migration of pancreatic ductal adenocarcinoma (PDAC) cells in vitro and it also markedly suppressed the tumor growth in a PDAC tumor xenograft model [173]. **Compound 12q** exhibited weak or no activity against other HDAC family members as well as 415 kinases, indicating good selectivity for SIRT-6 [100,173].

In the same cancer model, SIRT-6 inhibitors identified by a VS (see Section 3.6), having a quinazolinedione and salicylate-like structure, reduced proliferation and increased sensitivity to gemcitabine [174,175]. The most promising inhibitor with quinazolinedione structure showed a poor selectivity towards SIRT-1 and -2 [174]. Conversely, the family of SIRT-6 inhibitors with a salicylate-like structure, showed a selectivity ranging approximately from 10 to 30 folds towards SIRT-1 and -2 [175].

In addition, it was suggested that a synthetic lethal approach, enhancing DNA damage while concomitantly blocking SIRT-6-mediated repair responses through the use of SIRT-6 inhibitors, provides the rationale for the clinical evaluation of SIRT-6 inhibitors in the treatment of leukemia [176].

#### 2.6.2. SIRT-6 Modulators in Neurodegenerative Disorders

In PD, no studies in vitro or in vivo have been performed until now using an SIRT-6 modulator. However, it has been observed that in an MPTP-induced PD mouse model, brain-specific SIRT-6 knockout conferred neuroprotection, while SIRT-6 overexpression caused a more severe pathology to the mice. In the same study, Nicotine was proved to decrease SIRT-6 expression both in vitro and in vivo, and as a consequence to protect neurons from apoptosis [177]. In addition, inhibition of SIRT-6 was suggested to be a promising strategy to ameliorate PD and neurodegeneration, given that nicotine reduces the abundance of SIRT-6 in neuronal culture and brain tissue, this mediating the nicotine-induced neuroprotection [177].

One SIRT-6 inhibitor with quinazolinedione structure (named compound 1 and identified via in silico screening, see Section 3.6) [174] was administered following both a “preventive” and a “therapeutic” protocol in a mouse model of Experimental Autoimmune Encephalomyelitis (EAE), regarded as an MS animal model. No significant effects were obtained in the therapeutic protocol. Instead, SIRT-6 inhibition strikingly delayed EAE onset, impaired dendritic cell migration, downregulated pathogenic T cell inflammatory responses. Therefore, SIRT-6 inhibitors were suggested to represent novel therapeutic agents for the treatment of early stages of MS, or of other autoimmune disorders [178]. 

#### 2.6.3. SIRT-6 Modulators in T2D

SIRT-6 inhibitors identified by VS (see Section 3.6) in vitro induced GLUT1 upregulation and consequent augmented glucose uptake in L6 rat myoblasts and BxPC3 cells [179]. When tested in a murine model of T2D, one SIRT-6 inhibitor having a quinazolinedione scaffold, improved glucose tolerance and reduced plasma levels of insulin, triglycerides, and cholesterol [180].

The potential beneficial effect of using SIRT-6 inhibitors in T2D came also from an independent study, in which **Compound 6d** (a newly identified SIRT-6 inhibitor) was used in a mouse model of T2D and significantly increased the level of glucose transporter GLUT-1, thereby reducing blood glucose [181]. **Compound 6d** was identified by performing a screening study against an in-house chemical library containing about 2000 compounds by Fluor de Lys assay and showed good selectivity over other deacetylases including SIRT-1-3 and HDAC1-11 [181].

### 2.7. SIRT-7 Modulators as Therapeutics

SIRT-7 is localized in the nucleus, where it is involved in the activity of RNA polymerase I and it is important for cell viability [182,183]. Very few reports are available for this sirtuin. The interest in SIRT-7 has increased in the last 10 years, but a lot of work still needs to be accomplished to understand its role in the etiology of pathologies.

#### 2.7.1. SIRT-7 Modulators in Cancer

Zhang and colleagues showed that compounds **2800Z** and **40569Z** (identified by VS, see Section 3.7) are specific SIRT-7 inhibitors exerting an antiproliferative effect on liver cancer in vitro and in vivo [184]. 

In addition, Kim and colleagues identified compound **97491** as a specific SIRT-7 inhibitor able to induce apoptosis in uterine sarcoma [185]. This compound was identified by an in vitro enzyme activity assay using compounds obtained from the Korea chemical bank [185].

#### 2.7.2. SIRT-7 Modulators in Neurodegenerative Disorders

SIRT-7 possesses a major role in numerous neuronal pathways. For instance, it regulates rRNA synthesis and assembly of ribosomes via the changes in NAD^+^/NADH ratio [183] and promotes the repair mechanism of non-homologous DNA damage [186]. Nevertheless, there are no reports explaining its role in neurodegenerative diseases.

#### 2.7.3. SIRT-7 Modulators in T2D

The role of SIRT-7 in T2D is still obscure and there are no reports on the use of SIRT-7 inhibitors in this disease. Li and colleagues showed that the overexpression of SIRT-7 increases hyperglycemia and renal dysfunction in rats [187]. These data suggest that the use of SIRT-7 inhibitors could help T2D treatment.

### 2.8. Pan-Sirtuin Modulators as Therapeutics

Several pan-sirtuin modulators have been identified and assessed in different cellular and animal settings. The modulators identified have mainly an inhibitory action, since the discovery of activators is more complex and just a few SIRT activators have been discovered in the past decades. The main issues related to the use of non-specific sirtuin inhibitors are represented by the ineffectiveness of the treatment due to the inhibition of two or more sirtuins exerting an opposite effect in the cells [89], until further side effects on non-sirtuin targets are found, which may cause over-toxicity. Thus, in principle, it is preferable not to use pan-sirtuin modulators and, in case no other options are available, extra caution may be needed with these modulators.

#### 2.8.1. Pan-Sirtuin Modulators in Cancer

Cambinol (or NSC-112546) is an SIRT-1 and SIRT-2 inhibitor with equal efficiency (IC_50_-_SIRT1_ = 56µM, IC_50-SIRT2_ = 59 µM) and a less efficient SIRT-5 inhibitor (IC_50-SIRT5_ > 300 µM). Cambinol is a chemically stable compound related to splitomicin, able to reduce cell proliferation, migration, and invasion and can induce apoptosis and cell cycle arrest in lung cancer, lymphoma and cervical cancer [188], multiple myeloma [189], hepatocarcinoma, neuroblastoma, and breast cancer [190,191,192].

Tenovin-1 and -6 inhibit SIRT-1 and SIRT-2 and decrease cell growth in Burkitt’s lymphoma and melanoma cells [193], gastric cancer cells [194], and NSCLC [195]. In addition, promising results were obtained in decreasing tumor growth in leukemia and melanoma [113,196]. Tenovins were originally identified through a high-throughput screen designed to detect compounds that activate the tumor suppressor p53 [193].

Schnekenburger and colleagues showed that their newly synthesized **Compound 18** (identified via in silico screening, see Section 3.8) inhibits SIRT-1 and SIRT-2 with an anti-proliferative effect in glioma cells, both in in vitro and in vivo settings [197].

**Compound 3g**, designed by Laaroussi and colleagues, proved to be an SIRT-1 and SIRT-2 inhibitor with cytotoxic effects on leukemia, colorectal, lung and breast cancer cell lines [198].

**Sirtinol**, an SIRT-1 and SIRT-2 inhibitor, originally identified through a high-throughput, phenotypic screen in cells [199] determined a senescence-like growth arrest and decreased activation of the RAS-MAPK pathway in breast and NSCLC [200]. In breast cancer, Sirtinol induces cell death and destabilizes the Slug protein, thus antagonizing the effect on the metastasis capabilities of basal-like breast cancer [50,201]. In addition, **Sirtinol** enhanced chemosensitivity to camptothecin and cisplatin in the prostate and cervical cancer cells, resulting in a significant reduction in viable cells due to enhanced apoptotic cell death [200,202].

**Salermide** and its two analogs, **Compound 4b** and **6a**, identified by Rotili and colleagues applying hit-to-lead structural variations, showed a potent anti-proliferative effect on leukemia, lymphoma, colon, breast, NSCLC and glioblastoma cancer cells [90,203,204].

**BZD9L1** is another SIRT-1 and SIRT-2 inhibitor able to induce apoptosis in colorectal, leukemia, and breast cancer cell lines [205].

**Compounds 27** and **30**, discovered by Mellini and colleagues upon synthesis of a compound library of 30 pseudopeptides, were reported to inhibit SIRT-1, -2, and -3 and they are able to arrest cell growth in breast and lung cancer cells [54].

**JH-T4** inhibits SIRT-1, -2, and -3 and it has an antiproliferative effect on breast, colorectal, and lung cancer cells. JH-T4 was identified by carrying out structure–activity relationship studies based on the structure of TM, a thiomyristoyl lysine compound [206]. [206]

George and colleagues showed that the dual inhibitor of SIRT-1 and SIRT-3, **4-Bromo resveratrol**, obtained with a chemical modification from resveratrol [207] is able to reduce cell growth and induce apoptosis in melanoma cells in vitro and in vivo through metabolic reprogramming [208].

Hui and colleagues designed and synthesized a series of 2-(4-acrylamidophenyl)-quinoline-4-carboxylic acid derivatives as putative SIRT-3 inhibitors, the best of which is **Compound P6**, which acts also on SIRT-1 and SIRT-2. This compound is able to arrest the cell cycle of mixed-lineage leukemia (MLL) cells in G0/G1 phase [209].

Finally, nicotinamide, the pan-sirtuin inhibitor par excellence, exerts antiproliferative effects and induces apoptosis in leukemic, oral squamous cell carcinoma, lung, and prostate cancer cells [210,211,212,213].

#### 2.8.2. Pan-Sirtuin Modulators in Neurodegenerative Disorders

The only pan-sirtuin inhibitor reported in the literature with an effect on neurodegenerative disorders is nicotinamide, which seems to have the ability to restore cognitive deficit by reducing tau phosphorylation in mouse models of AD. The mechanism of action mediating the nicotinamide-induced beneficial effect is unknown. However, the increased expression of SIRT-2 with age suggests that the preferential target of nicotinamide in AD is SIRT-2 [214,215]. Nicotinamide was also evaluated in a transgenic mouse model of HD, and it caused the improvement of motor deficits [216]. The detailed mechanism of action was not investigated; nevertheless, the treatment with nicotinamide is able to increase the expression of brain-derived neurotrophic factor (BDNF) and peroxisome proliferator-activated receptor gamma coactivator 1-alpha (PGC-1α).

#### 2.8.3. Pan-Sirtuin Modulators in T2D

In INS-1 beta pancreatic cells, nicotinamide was reported to reduce the high glucose- and palmitate-induced cell death. The mechanism of action is not fully understood; however, the knockdown of SIRT-3 or SIRT-4 determines the same effect as nicotinamide treatment [217], suggesting that nicotinamide’s protection may be due to the inhibition of SIRT-3 and/or SIRT-4. Therefore, the use of inhibitors for these two sirtuins may represent a promising therapeutic approach.

## 3. Virtual Screening Strategies Guiding the Discovery of Sirtuins’ Modulators

During the last years, the rational design of novel bioactive compounds towards a specific biological target, such as enzymes and G-protein-coupled receptors (GPCRs), was deeply accelerated by computational strategies. In this context, the search of sirtuins’ modulators can be pursued, taking advantage of a consistent number of experimental data, such as X-ray crystallographic information, which turn in a viable support for drug design. In addition, collecting a number of known modulators allowed us to develop further ligand-based strategies, such as pharmacophore modelling, to support the preliminary evaluation of a high number of compounds, prior to VS.

Herein, we report and discuss the computational studies and VS approaches so far applied towards the discovery of novel sirtuin modulators, as described in the literature.

**Figure 2:**
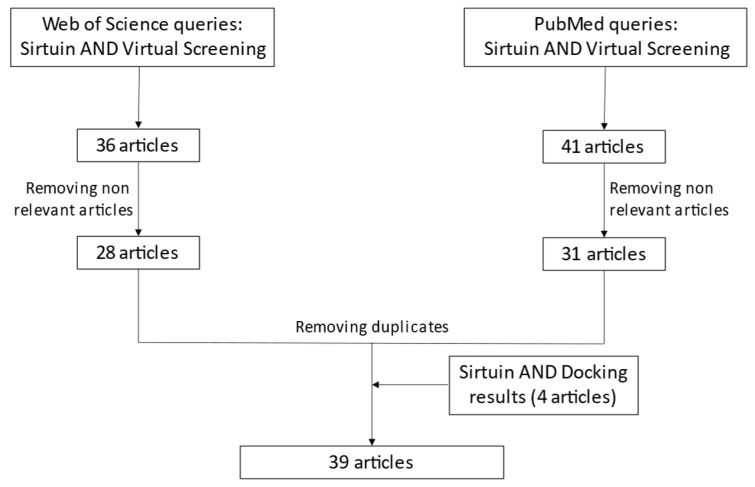
Workflow for article selection.

The analyzed articles were retrieved by means of a cross-search on PubMed [218] and Web of Science [219]. The queries combined the terms “sirtuin” AND “virtual screening” (VS). The merge of the two results produced as outcome a list of 77 articles, then refined to exclude non-relevant data (review articles, VS conducted on other targets, articles that do not contain VS, etc.) and duplicates. A similar method was followed using the queries “sirtuin” AND “docking” to include docking-based VS. Four articles were added. In total, 39 articles containing VS experiments applied to one or more sirtuins were obtained. Figure 2 summarizes the article selection procedure (last check 14-04-22), while Table 3 reports the applied computational methods and the obtained results.

### 3.1. Virtual Screening of SIRT-1 Modulators

To our knowledge, the first application of VS techniques to the discovery of SIRT-1 binders appears **in 2008**, when Huhtiniemi et al. performed a pharmacophore-based VS [220] using a previously reported structure-based model for SIRT-1 inhibitors [221]. A new oxadiazole-carbonylaminothiourea scaffold was individuated, and 47 analogues bearing this group were assessed in vitro against SIRT-1 and SIRT-2 [220]. The most potent compound showed a comparable potency with respect to the reference compound (an **EX-527 analogue**). **In 2009**, Sugunadevi Sakkiah et al. [222] explored the allosteric activation of SIRT-1 with a similar approach. In this case, the pharmacophore was modelled using three known SIRT-1 activators belonging to the imidazothiazoles class, and the best hypothesis was used to screen the Maybridge database [223]. Seven compounds were selected as promising hits. A few years later, the same group combined an analogous ligand-based pharmacophoric screening with the use of a Bayesian model [224]. A drug-like database was screened with both the models, and 16 novel candidates were selected as best hits based on the calculation of their energy gap (DFT application). A ligand-based (LB) pharmacophore model was also used **in 2015** by Vyas’ group [225] to screen the Zinc database [226] in search of new SIRT-1 activators. The model was generated using the structures of 10 known SIRT-1 activators, and top hits were submitted to docking in an HM of the target. A few years later, A. Azminah et al. combined the use of ligand-based and structure-based pharmacophoric models to identify novel SIRT-1 activators [227]. In particular, a structure-based pharmacophore was generated by the crystal structure of SIRT-1 co-crystalized with a small molecule sirtuin-activating compound (STAC, PDB code: 4ZZJ) [228]. The ligand-based model was instead built using 12 actives retrieved by the literature. A database of 1377 compounds by the HerbalDB [229] was screened with the two pharmacophore hypotheses. The ligand-based approach retrieved quinine, quinidine and gartarin as best candidates for SIRT-1 activation. The SB model individuated mulberrin as best hit. In vitro studies confirmed the bioactivity of these compounds. A similar approach was chosen by Pulla’s group [57] **in 2016**. In particular, an energy-based pharmacophore model was built on the crystal structure of SIRT-1 in complex with an **EX527 analogue** and NAD^+^ (PDB code: 4i5i) [230], while a ligand-based pharmacophore model was developed using a training set of 79 molecules. The pharmacophore features were used to pre-filter a large database from Asinex [231] and a smaller inhouse library. The resulting compounds were then subjected to molecular docking using a multi-step procedure. A benzimidazole derivative was identified as a promising scaffold on the basis of in vitro and in vivo screenings (see **Compound 17** mentioned in Section 2.2.1).

**In 2012**, Alvala et al. [52] performed an SBVS of an in-house database against a homology model of the SIRT-1 catalytic domain. A new isonicotinamidic scaffold was used, and three putative inhibitors were selected for in vitro testing. The positive outcome of the analysis allowed further optimization, leading to a new potent acridinedione with anticancer potential (as mentioned in Section 2.1.1). **In 2014** [232] the same group used a structure-based approach to identify novel SIRT-1 modulators. In particular, the crystal structure of SIRT-1 in complex with an analogue of **EX-527** (a SIRT-1 inhibitor) and NAD^+^ (PDB code 4I5I) [230] was used to screen the Asinex database [231], resulting in the individuation of two promising inhibitors. In the same article, a similar procedure was followed for the design of SIRT-1 activators, using a previously reported homology model [233] of the target as structural information. A new activator was proposed, and the compound efficacy was confirmed by in vitro tests. A few years later, Padmanabhan et al. [234] used the hSIRT-1 crystal structure [235] in complex with ADPR (pdb code: 4KXQ) to screen a Drug Bank [236] library containing 1716 compounds. Two scaffolds (diphenyl derivatives and oxycoumarin derivatives) were selected and submitted to MD simulation analysis. Four compounds resulted to inhibit SIRT-1 deacetylase activity in vitro. More recently, Wössner et al. [59] proposed an iterative in silico-in vitro screening procedure for the individuation of novel SIRT-1 inhibitors. Initially, an SBVS against the EX-527-SIRT-1-NAD^+^ complex [230] (PDB ID 4I5I) was performed. A first series of in vitro experiments validated the individuation of a thienopyrimidone with a thiocyanate moiety as promising scaffold. Based on this result, phenyl thiocyanates-containing compounds were selected by the Princeton BioMolecular Research Compound collection [237] and the resulting 113 thiocyanates were docked in the SIRT-1 active site. The in vitro test of the best docked compounds led to the individuation of an improved thienopyrimidone-thiocyanate. Interestingly, these compounds showed high selectivity towards SIRT-2 and -3. In addition, the compounds discovered showed anti-cancer activity in vitro (see Section 2.2.1).

A similar method was used by Tugba Ertan-Bolelli and Kayhan Bolelli [238] to identify novel hSIRT-1 activators. In particular, they performed an SBVS of a zinc [226] library on the SIRT-1 allosteric domain, individuating seven putative activators. Among them, acebutolol was selected for MD studies.

A less classic approach is reported in the study of Wang et al. [239], which developed an innovative sequence-based prediction model. This method relies on the idea that as the sequence determined the three-dimensional fold of the target, it can be correlated to the ligand bioactivities with no need for structural information. A general (target-unspecific) model was therefore developed with a training set of protein–ligand interactions, using a support vector machine (SVM) [240,241,242] approach. The model output was the prediction of unknown protein–ligand interactions using as input a protein sequence and a small molecule library. In the present case, a drug-like Specs database [243] and SIRT-1 sequence were used. Five novel inhibitors were individuated by in vitro tests, highlighting the success of this strategy. **In 2016**, Sun et al. [244] used Inductive logic programming (ILP) to screen a large database of compounds belonging to the Traditional Chinese Medicines-Taiwan database [245] and Traditional Chinese Medicine Integrated Database [246]. Briefly, the used ILP-based software combined both the input data (ligand information by the literature) and background knowledge to produce an inhibitor structure pattern, which was then used as a query for the screening. **In 2019**, a contest-based study was described by Chiba et al. [247]. Different groups were proposed to prioritize a subset of putative SIRT-1 inhibitors among a 2.5-million-compound database through different VS methods, with the aim to perform a comparative study. The experimental testing of approximately 50% of the proposed compounds led to the discovery of seven structurally distinct hits.

### 3.2. Virtual Screening of SIRT-2 Modulators

In **2008**, W. Sippl et al. performed a first example of VS on SIRT-2 [248], combining ligand-based and structure-based techniques. In particular, the Chembridge database [249] was initially filtered according to lead-like features and to similarity with respect to Cambinol, a known SIRT-2 inhibitor (see Section 2.2.1 and Section 2.8.1). The resulting compounds were then docked in the SIRT-2 catalytic pocket (crystal structure in the apo-form, 1J8F [6]), and compounds showing a H bond to Gln167 (a key residue) were retained. Compounds with a low logP were prioritized, and the resulting molecules were submitted to in vitro validation. Five barbiturate and thiobarbiturate derivatives were proposed. The authors further optimized this scaffold in **2012** through a similar multi-step VS procedure [250]. Most active thiobarbiturates from the initial work were selected, and their fingerprints were used as queries to screen the Chembridge database [249]. The results were filtered according to drug-like properties and docked in the apo-SIRT-2 active site [6] (PDB code 1J8F). Top hits were subjected to additional in silico and in vitro studies, highlighting an ameliorate potency with respect to the previously proposed analogues. A similarity-based approach was again followed by the same group to design novel splitomicin-related SIRT-2 inhibitors [105]. The authors performed an SAR study on the splitomicin scaffold and reported the best activity for β-aryl derivatives of splitomicin. The fingerprints of the most active compounds were used as queries to screen the Chembridge [249] database. The top-ranked compounds were docked in the apo form of SIRT-2 [6] (PDB code 1J8F) and four candidates were evaluated in vitro (the best inhibitors, namely, **8c** and **(R)-8c**, showed potent anti-cancer effects, see Section 2.2.1). The outcomes revealed that the substitution of the original lactone with a lactam is well tolerated.

A pure structure-based technique **was instead** chosen by P. Sivaraman et al. [251], which virtually screened an NCI [252] Diversity Set using the apo-form of SIRT-2 (PDB code 1J8F) [6] as a docking template. Every ligand was submitted to 150 separate docking calculation, using the Lamarckian genetic algorithm local search (GALS) method: the system generates a set of solution and propagates the most suitable answers to the following generations. The top hits were tested in vitro, revealing a new potent nucleoside-like inhibitor of SIRT-2. In **2016**, Sacconnay et al. [253] performed an SBVS against the ADPR-bound form of SIRT-2 (PDB code 3ZGV) [254] in search of new SIRT-2 inhibitors. A 197,477-compound library from Specs was employed to this aim. The top ranked compounds were filtered according to drug-like properties and submitted to a cluster analysis. The best ranked compounds of each cluster were purchased and evaluated in vitro, leading to the identification of two novel scaffolds. In particular, the 5-benzylidene-hydantoin was reported to be a promising hit with anti-cancer potential.

In some cases, SB screening has been coupled with MD to generate suitable conformation of the target. This requirement has been dictated by the unavailability of an inhibitor-bound form of SIRT-2 until 2015 [112] (PDB code 4RMG). In **2004**, Tervo et al. [255] generated an artificial SIRT-2 conformation by submitting to MD the apo-form of SIRT-2 [6] (PDB code 1J8F) and calculating the average structure on the equilibrated portion of the dynamics. The generated construct exhibited an enlargement of the binding pocket, whose interaction fields and properties were computed. The Maybridge database [223] was then screened according to these properties (hydrophobicity, shape, favourable interactions with polar probes). The most promising compounds were submitted to docking in SIRT-2 structure, and the compounds with a similar pose and key interactions with respect to sirtinol were retained. Five compounds resulted to be active against SIRT-2 by in vitro tests. The same authors used a similar approach [256] again in **2006**. After the generation of the MD conformation, the docking of a known inhibitor was performed, identifying key interactions for binding. According to this analysis, two queries were developed and used to screen the Maybridge Screening Collection [223] and the Lead Quest databases. Four out of the eleven tested compounds resulted to be active in vitro at the micromolar concentration. One of these compounds bears a new scaffold, not previously reported in relation to SIRT-2 inhibition, featuring the indole ring as the main core of the inhibitor. In **2012** [257], Sakkiah et al. generated an inhibitor-bound conformation by docking sirtinol in the NAD^+^ binding site of SIRT-2 apo-form [6] (PDB code 1J8F) and submitting the complex to 5-ns MD simulation. Three different representative conformations of the protein were obtained by means of a cluster analysis. They were merged together to form a single dynamic pharmacophore model, which was then used to screen the CHEMDIV database [258]. According to their drug-likeliness, ADMET properties and docking analysis, the authors proposed 21 leads as putative SIRT-2 inhibitors. **In the same year**, the same group combined this coupled MD-SB approach with ligand-based techniques [259]. In particular, a pharmacophore model built on a training set of 19 inhibitors was used to screen various databases (NCI [252], Maybridge [223] and Chembridge [249]), and the best candidates were filtered according to drug-like and calculated ADMET parameters. Then, an MD-derived structure of the sirtinol-SIRT-2 complex was used as a docking template to refine the results. The candidates with a comparable or higher score with respect to known inhibitors of SIRT-2 were retained, prioritizing the poses which established significant H-bonds. Twenty-nine candidates were individuated. **In 2019**, a similar approach was used by Eren et al. [97]. An LB pharmacophore was developed starting from a series of 31 SirReal analogues and applied to screen a 13-million-compound ZINC drug-like database. The retrieved molecules were then submitted to docking, this time in the finally available inhibitor-bound form of SIRT-2 (SIRT-2-SirReal analogue-NAD^+^, PDB code: 5DY4) [260]. The Glide [261,262] Virtual Screening Workflow (VSW) was used to this aim. The MM-GBSA technique was applied to increase the accuracy of the predicted pose. According to a PCA-based cluster analysis, 31 compounds were selected for in vitro evaluation. Two compounds showed good in vitro inhibitory potency and a certain degree of selectivity toward SIRT-1,-3,-5 (see Section 2.2.2). 

**In 2021, Kessler et al.** chose a machine learning approach with the aim to identify new selective inhibitors of SIRT-2 [263]. In particular, a Multilayer Perceptron (MLP) model [242] was built using a set of 234 inhibitors of SIRT-2 and 234 putative inactive molecules. The model was applied to screen the ZINC [226] collection of FDA-approved drugs, in a drug-repositioning perspective. The ML approach was then validated in silico through docking in SIRT-2 crystal structure (5YQL) [99]. The study reveals a promising ligand-based technique, which may be applied to other targets, even in the absence of a target 3D structure. **In the same year**, Khanfar and Alqtaishat [264] reported an interesting combination of pharmacophore-based screening and QSAR techniques. An ensemble of structure-based pharmacophoric models was built using a set of 18 SIRT-2 co-crystals. Nineteen models were regarded as satisfying by means of ROC curves [265] analysis. In parallel, a set of SIRT-2 inhibitors was submitted to the calculation of their physicochemical descriptors. Genetic Function Algorithm [266] and Multiple Linear Regression analysis [267,268,269] were used to select the best combination of pharmacophores and descriptors, building a single mathematical equation able to predict and explain activities. In particular, the resulting equation reports three descriptors and the fit value of the most suitable pharmacophore model. The latter was used to screen the AnalytiCon Discovery database of purified natural products [270]. The matching compounds were ranked according to their predicted IC_50_, according to the integrated QSAR model. Among the 10 bioassayed compounds, two candidates (asperphenamate and salvianolic acid B) showed inhibitions with IC_50_ in the low micromolar concentration.

**Table 3:** Perspective of the computational studies leading to the identification of selective and/or pan-Sirtuins modulators (shown in green). The chemical structure and the explored biological activity of the discovered hit compounds are reported. The applied virtual screening (VS) strategy is specified as structure-based (SBVS) or ligand-based (LBVS) methodology. The results are listed based on the sirtuin type (alternatively in gray and cyan), according to SBVS followed by LBVS and combined SB-LB approaches, as chronological order. Data about parasitic sirtuins (depicted in coral) are also detailed, referring to the Leishmania (Lm-Sirt), *Trypanosoma cruzi* (Tc-Sirt) and *Schistosoma mansoni* (Sm-Sirt) sirtuins.

Year	Ref.	Title	SIRT(s)	Type of VS	Notes	Selectivity Over Other Isoforms	Experimental Validation	Screened Database (n. of Compounds)	Software(s)	Most Active Compound/Proposed Compound	Activator/Inhibitor	Potency [n. of Proposed Compounds by Computational Study]
2008	[220]	Oxadizole-carbonylaminothioureas as SIRT-1 and SIRT-2 inhibitors	**1**	**SBVS**	Pharmacophore based	1,2	YES	Maybridge and Leadquest libraries	Unity 4.3.1/Sybyl 7.1	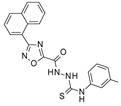 oxadizole-carbonylaminothiourea	I	13 μM (IC_50_, SIRT-1)
2012	[52]	Novel acridinedione derivatives: design, synthesis, SIRT-1 enzyme and tumor cell growth inhibition studies.	**1**	**SBVS**	Target: HM	Not tested	YES	In house database (2500)	Glide, Gold, AutoDock 4.0	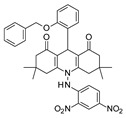 acridinedione derivatives	I	0.25 μM (IC_50_)
2014	[232]	Structure-based drug design of small molecule SIRT-1 modulators to treat cancer and metabolic disorders	**1**	**SBVS**	Target model for inhibitors: crystal structureTarget model for activators: HM of the allosteric site	Not tested	YES	Asinex (>600000)	Glide 5.0	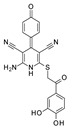	I, A	16.35 μM (IC_50_)
2016	[234]	Identification of New Inhibitors for Human SIRT-1: An in-silico Approach	**1**	**SBVS**		Not tested	YES	Drug bank library from ZINC (1716)	AutoDock Vina 1.1.2	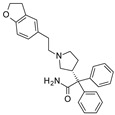 diphenyl and oxycoumarin derivatives	I	77.7% inhibition @5μM
2020	[59]	Sirtuin 1 Inhibiting Thiocyanates (S1th)-A New Class of Isotype Selective Inhibitors of NAD(+) Dependent Lysine Deacetylases	**1**	**SBVS**	Iterative in vitro-in silico screenings	2,3,5	YES	Small library of previously identified putative SIRT-1 inhibitors	GOLD 5.6	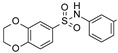 Thiocyanates	I	5.2 μM (IC_50_)
2021	[238]	In Silico Design of Novel SIRT-1 Enzyme Activators for the Treatment of Age-related Diseases and Life Span	**1**	**SBVS**		Not tested	NO	Zinc (150 000)	Information not available	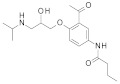 acebutolol and others	A	NC [7]
2009	[222]	Pharmacophore Mapping and Virtual Screening for SIRT-1 Activators	**1**	**LBVS**	Pharmacophore based	Not tested	NO	Maybridge	HipHop module/CATALYST	isothiazole scaffold benzimidazole scaffold	A	NC† [7]
2014	[224]	Theoretical approaches to identify the potent scaffold for human SIRT-1 activator: Bayesian modeling and density functional theory	**1**	**LBVS**	Bayesian model, pharmacophore model	Not tested	NO	Maybridge (60,000), Chembridge (50,000), NCI (200,000), and ChemDiv (700 000)	Discovery Studio v 3.1	Various	A	NC [16]
2016	[244]	Ligand-based virtual screening and inductive learning for identification of SIRT-1 inhibitors in natural products	**1**	**LBVS**	Inductive logic programming	Not tested	NO	Traditional Chinese Medicines-Taiwan Database and Traditional Chinese Medicine Integrated Database (1 444 880)	DMax Chemistry Assistant software	Various	I	NC [3]
2015	[225]	Ligand and structure-based approaches for the identification of SIRT-1 activators.	**1**	**LB-SB**	LBVS: Pharmacophore basedSBVS Target model: HM of Sirt-1	Not tested	NO	ZINC database	DISCOtech, GASP/SYBYL X 1.2 (pharmacophore)Surflex-Dock/SYBYL X 1.2 (docking)	Various	A	NC [2]
2016	[57]	Energy-Based Pharmacophore and Three-Dimensional Quantitative Structure--Activity Relationship (3D-QSAR) Modeling Combined with Virtual Screening To Identify Novel Small-Molecule Inhibitors of Silent Mating-Type Information Regulation 2 Homologue 1 (SIRT-1).	**1**	**LB-SB**	LB and SB pharmacophore models combined with docking-based VS	Not tested	YES	ASINEX (5 000 000), in-house (971)	PHASE 3.4/Maestro 9.3 (pharmacophore) Glide 5.8/Maestro 9.4 (docking)	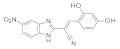 benzimidazole derivative	I	4.34 μM (IC_50_)
2019	[227]	In silico and in vitro identification of candidate SIRT-1 activators from Indonesian medicinal plants compounds database	**1**	**LB-SB**	LB and SB pharmacophore models	Not tested	YES	Indonesian Herbal Database (1377)	LigandScout 4.2	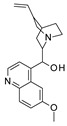 mulberrin, quinine, quinidine, and gartanin	A	1.14 μM (EC_50_)
2011	[239]	Computational screening for active compounds targeting protein sequences: methodology and experimental validation.	**1**	**Other**	Sequence-based VS	Not tested	YES	SPECS drug-like library (85 000)	LIBSVM	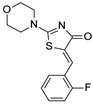 various	I	5.72 μM (IC_50_)
2019	[247]	A prospective compound screening contest identified broader inhibitors for SIRT-1	1	Other	Contest-based approach	Not tested	YES	ENAMINE (2 459 912)	various	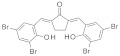 various	I	4.1 μM (IC_50_)
2004	[255]	An in silico approach to discovering novel inhibitors of human SIRT-2	**2**	**SBVS**	Queries: (SB) features calculated on MD generated conformation + Docking	Not tested	YES	Maybridge	Unity 4.3.1/Sybyl v6.8 (feature-based), GOLD v1.2 (docking)	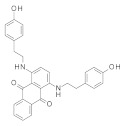 Phenol derivatives	I	56.7 μM (IC_50_)
2006	[256]	Discovering inhibitors of human SIRT-2: Novel structural scaffolds	**2**	**SBVS**	Queries: (SB) features calculated on MD generated conformation + Docking	Not tested	YES	Maybridge Screening Collection and LeadQuest libraries	Unity 4.4/Sybyl 6.9 (feature-based), GOLD 2.0 (docking)	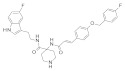 indole derivatives, phenol derivatives	I	51 μM (IC_50_)
2010	[251]	Design of a novel nucleoside analog as potent inhibitor of the NAD+ dependent deacetylase, SIRT-2.	**2**	**SBVS**		1	YES	NCI Diversity Set II	AutoDock 4.0	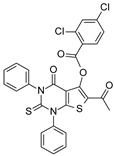 nucleoside analog (thieno [2,3-d]pyrimidine)	I	8.7 μM (IC_50_)
2012	[257]	Molecular Docking and Dynamics Simulation, Receptor-based Hypothesis: Application to Identify Novel SIRT-2 Inhibitors	**2**	**SBVS**	SBVS pharmacophoric model based on MD-generated conformations + docking	Not tested	NO	CHEMDIV database (700 000)	Ligand Pharmacophore Mapping/DS (screening), LigandFit/DS and GOLD (docking)	Various	I	NC [21]
2016	[253]	5-Benzylidene-hydantoin is a new scaffold for SIRT inhibition: From virtual screening to activity assays.	**2**	**SBVS**		1	YES	Specs library (197 477)	GOLD v5.2	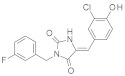 5-Benzylidene-hydantoin	I	37.7 μM (IC_50_)
2008	[248]	Thiobarbiturates as Sirtuin Inhibitors:Virtual Screening, Free-Energy Calculations, and Biological Testing	**2**	**LB-SB**	LB: similarity based	1	YES	Chembridge database (∼328 000)	MOE (fingerprints) GOLD 3.2 (docking)	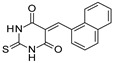 thiobarbiturates	I	9.1 μM (IC_50_)
2008	[105]	Structure-activity studies on splitomicin derivatives as sirtuin inhibitors and computational prediction of binding mode	**2**	**LB-SB**	LB: similarity based	Not tested	YES	Chembridge (∼328 000)	MOE (fingerprint), GOLD 3.0 (docking)	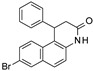 lactame analogues of beta-Phenylsplitomicins	I	6.4 μM (IC_50_)
2012	[259]	Pharmacophore modeling and molecular dynamics simulation to identify the critical chemical features against human SIRT2 inhibitors	**2**	**LB-SB**	LB: pharmacophore basedSB: VS on MD-derived protein conformation	Not tested	NO	NCI (5672), Maybridge (26 490), Chembridge (17 885)	Discovery Studio v2.5 (pharmacophore), GOLD (SB)	Not reported	I	NC [29]
2012	[250]	Binding free energy calculations and biological testing of novel thiobarbiturates as inhibitors of the human NAD(+) dependent histone deacetylase SIRT-2	**2**	**LB-SB**	LB: similarity-based	Not tested	YES	Chembridge	MOE (fingerprint), GOLD 4.0 (SBVS)	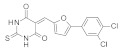 Thiobarbiturates	I	1.5 μM (IC_50_)
2019	[97]	Pharmacophore modeling and virtual screening studies to identify novel selective SIRT-2 inhibitors	**2**	**LB-SB**	LB: Pharmacophore-based	1,3,5	YES	ZINC drug-like database (13 000 000)	Schrodinger Small-Molecule Drug Discovery Suite (pharmacophore) Glide (SBVS)	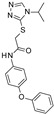 various (triazines and dimethylfuran derivatives among others)	I	84.28% inhibition @300μM
2021	[264]	Discovery of Potent Natural-Product-Derived SIRT-2 Inhibitors Using Structure- Based Exploration of SIRT-2 Pharmacophoric Space Coupled With QSAR Analyses.	**2**	**LB-SB**	SBVS pharmacophoric model combined with (SB+LB) QSAR	Not tested	YES	AnalytiCon Discovery database of purified natural products (5637)	DISCOVERY STUDIOv2.5	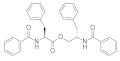 asperphenamate and salvianolic acid B	I	1.94 μM (IC_50_)
2021	[263]	Targeting Epigenetic Regulators Using Machine Learning: Potential SIRT-2 Inhibitors	**2**	**LB-SB**	LB: Machine learning model	Not tested	NO	ZINC/FDA library	WEKA (ML), AutoDock Vina/PyRx (SBVS)	Various	I	NC [43]
2015	[271]	Virtual screening approach of sirtuin inhibitors results in two new scaffolds	**3**	**SBVS**		1,2	YES	ZINC database (> 8 000 000)	Glide v5.8	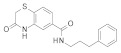 3-oxo-1,4-benzothiazinyl compounds and 4-(1-piperidyliminomethyl)benzene-1,3-dioles	I	40% inhibition @200 μM
2013	[272]	Identification of novel SIRT-3 inhibitor scaffolds by virtual screening	**3**	**LB-SB**	LB: similarity based	1,2	YES	Chembridge EXPRESS-Pick Collection database	Phase 3.2/ Schrodinger (similarity) + Glide 5.7 (SBVS)	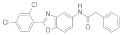 benzoxazoles, furopyrimidines	I	71% inhibition (200 μM)
2021	[143]	Structure-Guided Design of a Small-Molecule Activator of SIRT-3 that Modulates Autophagy in Triple Negative Breast Cancer	**3**	**SBVS**	SB compound optimization	Not tested	YES	ZINC database (> 8 000 000)	Glide	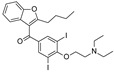 ZINC03830212	A	EC_50_ = 3.25 μM
2017	[273]	Molecular modeling, dynamics studies and density functional theory approaches to identify potential inhibitors of SIRT-4 protein from Homo sapiens: a novel target for the treatment of type 2 diabetes.	**4**	**SBVS**	Target: HM	Not tested	NO	LifeChem, Specs, ZINC and MayBridge libraries	Glide/Maestro/Schrodinger v10.1	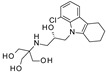 Indole derivative	I	NC [1]
2018	[274]	Structure-based discovery of new selective small-molecule SIRT-5 inhibitors	**5**	**SBVS**	SBVS + protein–ligand interaction fingerprint (IFP)-based filter	2,6	YES	In-house database (>15,000)	AutoDock Vina	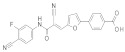 (E)-2-cyano-N-phenyl-3-(5-phenylfuran-2-yl)acrylamide derivatives	I	5,59 μM (IC_50_)
2014	[179]	Discovery of Novel and Selective SIRT-6 Inhibitors	**6**	**SBVS**	Target: modified structure of Sirt-6	1,2	YES	ASINEX subset of CoCoCo database	Glide v.5.8	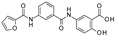 salicylate-based derivative	I	89 μM (IC_50_)
2015	[174]	Quinazolinedione SIRT-6 inhibitors sensitize cancer cells to chemotherapeutics	**6**	**LB-SB**	LB: pharmacophore, similarity based	1,2	YES	CoCoCo database	Instant JChem (Chemaxon) ver. 5.11.5	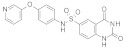 **Compound 1** as Quinazolinedione prototype	I	106 μM (IC_50_)
2018	[170]	Identification of a cellularly active SIRT-6 allosteric activator	**6**	**SBVS**	Allosteric site search + docking	Not tested	YES	Various DB (> 5,000,000)	GLIDE software (Schrödinger suite 2009, v5.5)	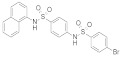 AN-988/40889624	A	EC_50_ = 173 μM
2020	[173]	Discovery of Potent Small-Molecule SIRT-6 Activators: Structure–Activity Relationship and Anti-Pancreatic Ductal Adenocarcinoma Activity	**6**	**SBVS**	Allosteric site search + docking	Not tested	YES	Specs, ChemDiv, Selleck, and MedChemExpress and in-house database	GOLD software	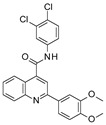 HIT20	A	147.60 (% Peptide Demyristoylation Activation @20 μM)
2021	[275]	Screening of SIRT-6 inhibitors and activators: A novel activator has an impact on breast cancer cells	**6**	**LB-SB**	LB: pharmacophore, similarity based	1,2	YES	ENAMINE (4 103 115), Chembridge (1 022 400), in house library of 1,4-dihydropyridine derivatives (∼100)	MOE (pharmacophore, similarity, docking), Glide/Maestro/ Schrodinger (docking)	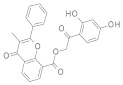 4H-chromen analogs (A), 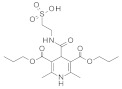 1,4-dihydropyridine (I)	A, I	80 μM (EC_50_, activator) 60% @200 μM (IC_50_, inhibitor)
2022	[184]	Discovery of SIRT-7 Inhibitor as New Therapeutic Options Against Liver Cancer	**7**	**SBVS**	Target: protein modeling via fold recognition	1,6	YES	Chemdiv database (939319)	AutoDock Vina	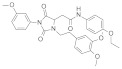 **Compound 2800Z**	I	Inhibition of SIRT-7 deacetylase activity
2011	[276]	Structure-based development of novel sirtuin inhibitors	**2,3,5,6**	**SBVS**		2,3,5,6	YES	NCI diversity set (1990)	AutoDock Vina	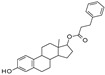 SIRT-2 specific: tetracyclic compounds	I	4.8 μM (IC50, SIRT-2)
2017	[197]	Discovery and Characterization of R/S-N-3-Cyanophenyl-N’-(6-tertbutoxycarbonylamino-3,4-dihydro-2,2-dimethyl-2H-1-benzopyran-4- yl)urea, a New Histone Deacetylase Class III Inhibitor Exerting Antiproliferative Activity against Cancer Cell Lines	**1,2**	**SBVS**		1,2,3	YES	In-house library (17)	AutoDock Vina	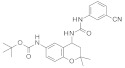 **Compound 18**		6.2 μM (IC_50_, SIRT-1) 4.2 μM (IC_50_, SIRT-2)
2018	[277]	Identification of Bichalcones as Sirtuin Inhibitors by Virtual Screening and In Vitro Testing	**1,2**	**SBVS**	SBVS on different conformations of sirt-1 and sirt-2	3	YES	pan-African Natural Products Library (463)	GOLD, Glide/Maestro/ Schrödinger v5.8	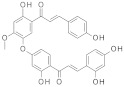 Bichalcones	I	40.8 μM (IC50, SIRT-1)
2019	[278]	Structure-based identification of novel sirtuin inhibitors against triple negative breast cancer: An in silico and in vitro study	**1,2,3,4,5,6,7**	**SBVS**		Not applicable	YES	Plant-derived inhibitors (24), synthetic inhibitors (3) with reported epigenetic modulatory and anticancer potential, PubChem	Glide/Maestro/ Schrödinger	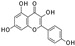 Sulforaphane (SIRT-1,-5), Kaempferol (SIRT-3) Apigenin (SIRT-6)	I	12,5 μM (IC50)
2021	[279]	In silico Repurposing of Drugs for pan-HDAC and pan-SIRT Inhibitors: Consensus Structure-based Virtual Screening and Pharmacophore Modeling Investigations	**1,2,3,5,6**	**SBVS**	Consensus SBVS on sirt-1,2,3,5,6 with 3 software	Not applicable	NO	(FDA)-approved drugs (1502)	Glide, FRED v3.3.1.2, AutoDock Vina/PyRx v1.1.2	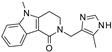 Alosetron, cinacalcet, indacaterol (virtual pan-SIRT I)	I	NC [3]
2008	[280]	Structure function analysis of Leishmania sirtuin: An ensemble of In silico and biochemical studies	**Lm Sir2**	**LB-SB**	LB: Fingerprint basedSBVS target: LmSir2 HM	2	YES	National Cancer Institute (NCI) 3D database (~200 000)	MOE, FlexX/SYBYL 6.9	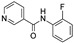 Nicotinamide derivative	I	1,49 mM (IC50)
2012	[281]	Anti-*Trypanosoma cruzi* activity of nicotinamide	**Tc Sir2**	**LB-SB**	LB: fingerprint based (nicotinamide)SBVS target: TcSir2 HM	Not tested	YES	Zinc database	MolDock	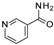 Nicotinamide	I	~100 μM (IC50)
2014	[282]	Computational Studies on Sirtuins from *Trypanosoma cruzi*: Structures, Conformations and Interactions with Phytochemicals	**Tc Sir2**	**SBVS**	Targets: 2 HM of the 2 Tc sirtuins (closed state)	2,5	NO	Phytochemicals with antitrypanosomal activity collected by the literature (50)	GOLD v5.1	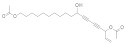 Anacardic acid derivative, aculeatin D, 16-acetoxy-11-hydroxyoctadeca-17-ene12,14-diynylethanoate, vismione D	I	NC [4]
2016	[283]	In-silico analysis of SIRT-2 from *Schistosoma mansoni*: structures, conformations, and interactions with inhibitors	**Sm Sir2**	**SBVS**	Target: SmSir2 HM	2	NO	ZINC derived database (18 560)	idock	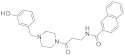 Phenol derivative	I	NC [4]

### 3.3. Virtual Screening of SIRT-3 Modulators

In **2013**, H.S. Salo [272] et al. developed a combined LB/SB workflow for the discovery of SIRT-3 inhibitors. The group previously identified some thioacetyl-lysine-containing SIRT-3 inhibitors [284]. The shape and some pharmacophoric features of these molecules were used as a query to pre-filter the Chembridge [249] EXPRESS-Pick Collection database. The obtained small molecules were docked in the active site of SIRT-3 [285] (3GLR), and top hits were selected for in vitro evaluation. Two novel scaffolds were individuated and the 5-amino-2-phenyl-benzoxazole one was selected for further studies. **Some years later** [271], the same group performed again a VS toward this target with the aim to individuate a putative binding site that may be exploited for the design of allosteric inhibitors. The crystal structure of SIRT-3 bound with a reaction intermediate (PDB ID: 3GLT) [285] was submitted to this computational search [286]. The ZINC [226] database (over 8 million compounds) was then screened with the VSW procedure with Glide [261,262]. The presented strategy produced three moderately potent sirtuin inhibitors, which shows a certain selectivity toward SIRT-2 and SIRT-1. **In 2021**, exploring the X-ray crystallographic data of SIRT-3 (PDB code = 4FVT) [287] allowed to screen in silico 1.4 million small-molecule compounds, leading to the hit compound ZINC03830212 as an SIRT-3 modulator. This derivative has been modified based on structure-guided studies towards the development of more effective compounds featuring anti-cancer ability, such as **Compound 33c** [143] (see the previous Section 2.3.1).

### 3.4. Virtual Screening of SIRT-4 Modulators

To individuate new inhibitors of SIRT-4, a VS campaign was carried out by Choubey et al. [273], together with docking experiments. Quantum Polarized Ligand Docking [288] was employed to optimize the geometry of the docked compounds. Three ligands were selected for binding free energy evaluation and stereoelectronic analysis. An indole derivative was proposed as lead molecule, according to the in silico evaluated parameters.

### 3.5. Virtual Screening of SIRT-5 Modulators

**In 2018**, Liu et al. performed an SBVS approach to discover new inhibitors of SIRT-5 [274]. The crystal of SIRT-5 in complex with a bicyclic intermediate [289] (4F56) was chosen as a docking template to screen an in-house library of more than 15,000 compounds. An interaction fingerprint analysis involving several protein–ligand interactions was carried out on the best docked compounds. As Tyr102 and Arg105 were individuated as exclusive residues of SIRT-5, being also key residues for the recognition of acidic acyl-lysine substrates [22,289,290], the compounds which showed H-bonds or electrostatic interactions with these amino acids were selected as potentially selective hits. After visual inspection, 20 candidates were evaluated in vitro.

### 3.6. Virtual Screening of SIRT-6 Modulators

**In 2014**, a structure-based screening focused on the SIRT-6 X-ray crystallographic structure (PDB code = 3K35) [291] and the CoCoCo database [292] led to the discovery of selective SIRT-6 inhibitors bearing the salicylate-like structure [179]. These derivatives were further optimized by Sociali et al. in 2015 [174]. In particular, different substructure searches were performed against the CoCoCo database [292], with the aim to fulfill the structural requirements exhibited by the previously identified SIRT-6 modulators [179], and to find more potent analogues. As a result, **Compound 1** has been identified and described for the treatment of cancer, MS and T2D (see Section 2.6) [174].

More recently, in Tenhunen et al. [275] (**2021**), a further combination of SB and LB techniques was pursued towards the identification of novel SIRT-6 modulators. Enamine [293] and Chembridge databases [249] were screened according to a ligand-based pharmacophore model built on a small set of known Sirtuin modulators, such as **resveratrol**, **Ex-527**, **sirtinol**, and **quercetin**, taking into account as reference compounds those previously described by Parenti and coll [179].

The obtained hits from both the procedures together with a small library of synthetic 1,4-dihydropyridine derivatives were docked in SIRT-6 active site [291] (3K35). In vitro tests individuated 4H-chromen as SIRT-6 activator and a novel inhibitor with a 1,4-dihydropyridine scaffold. 1,4-dihydropyridines were already reported in relation to SIRT-1 [294,295].

**In 2018**, Huang et al. combined in silico studies and experimental approaches to individuate novel SIRT-6 activators [170]. By using the Allosite method developed by the same group [296], the putative SIRT-6 allosteric site was individuated and used to screen a library of more than 5,000,000 compounds. Among the (20) selected and purchased molecules, Huang et al. identified two hits, **AN-988/40889624** and **AH-487/41802661**, featuring halfmaximal effective concentration (EC_50_) values of 173.8 ± 1.3 μM and 217.6 ± 1.1 μM (mean ± s.d.) acting as SIRT-6 activators (see Section 2.6.1). More recently, **in 2020**, compound **Hit20** was also discovered as SIRT-6 activator, thanks to molecular docking adopted to screen commercial chemical databases including Specs, ChemDiv, Selleck, and MedChemExpress, as well as an in-house database (see Section 2.6.1) [173]. The prototype has been then further optimized towards the lead analogue **compound 12q**.

### 3.7. Virtual Screening of SIRT-7 modulators

The search for putative SIRT-7 modulators via in silico screening or structure-based studies is hampered by the absence of complete X-ray crystallographic data of the enzyme, in the presence or not of ligand. Indeed, the crystal structure of the only N-terminal domain of the protein is available (PDB code = 5IQZ) [297], making, so far, VS campaigns quite complicated.

On the other hand, no ligand-based approaches have been attempted, probably because of the limited number of known selective SIRT-7 modulators.

Very recently (**2022**), Zhang et al. described a predicted model of the SIRT-7 structure, by using the fold recognition (or threading) method, followed by structure-based VS [184]. The structural model of the human SIRT-7 was managed by I-tasser [298] following a procedure described in the literature [299,300]. Subsequent VS screening was carried out by Autodock Vina [301] by taking into account the Chemdiv database [258] containing 939,319 structurally diverse compounds.

The study led to the selective SIRT-7 inhibitors **2800Z** and **40569Z**. These compounds exhibit high affinities toward SIRT-7 and low affinities to other SIRT proteins, and were shown to inhibit SIRT-7 deacetylation activity in vitro [184]. The related potential role in cancer has been investigated (see Section 2.7.1)

### 3.8. Pan-Sirtuin Modulators

With the aim to find isoform-specific compounds, Schlicker et al. [276] screened the National Cancer Institute (NCI) [252] diversity set against the crystal structures of SIRT-2 [6], -3 [285], -5 [302] and -6 [291]. The structures were modified in silico to accommodate an ADPR molecule, using as a reference structure the SIRT-6 complex (PDB code: 3K35) [291]. The top compounds from each screening were selected for experimental validation and isoform selectivity evaluation. Among the 20 active compounds, 14 compounds had selectivity toward SIRT-2, while no isoform-selective modulators were retrieved for the other sirtuins. On the opposite, multiple sirtuin VS was applied to individuate non-selective/pan-sirtuins inhibitors, which proved to be beneficial in some cancer conditions [303,304].

**In 2017**, Schnekenburger et al. identified **Compound 18** as an SIRT-1/-2 modulator. The study involved a molecular docking step, useful to compare the predicted binding affinities of the candidates. The in-house library was screened against the X-ray crystal structure of SIRT-1 (PDB code = 4I5I) [230] and SIRT-2 (PDB code = 4RMG) [112], using AutoDock Vina [301]. The compound showed an anti-proliferative effect in glioma cells, both in in vitro and in vivo settings (see Section 2.8.1) [197].

**In 2018**, Karaman et al. [277] performed a consensus SBVS to identify new SIRT-1 and -2 inhibitors. In particular, the p-ANAPL database [305] was prefiltered to exclude PAINs [306] and submitted to docking against two SIRT-1 [228,230] and four SIRT-2 [112,307,308,309] crystal structures (PDB IDs SIRT-1: 4I5I, 4ZZJ; SIRT-2: 4R8M, 4L3O, 4RMH, 5D7P). Top-scored compounds by the two tranches were combined and the duplicates were removed. Seven compounds were tested in vitro on SIRT-1, SIRT-2 and SIRT-3, and two bichalcones were individuated as moderately active inhibitors. **More recently**, a repositioning study [279] was performed to individuate (pan-)inhibitors of SIRT-1 (4I5I) [230], -2 (5DY4) [260], -3 (4BV3) [310], -5 (5XHS) [290] and -6 (6HOY) [311]. In particular, a set of 1502 FDA-approved drugs was screened against the crystal structures of the targets using three different pieces of software (Glide [261,262], FRED [312], and AutoDock Vina/PyRx [313]). A consensus score was produced by calculating the average value of the three scores. **Alosetron**, **cinacalcet**, and **indacaterol** were individuated as the best pan-SIRT putative inhibitors. **In 2019**, Sinha et al. [278] performed an SBVS of plant-derived inhibitors against multiple sirtuin isoforms (SIRT-1 PDB:4I5I [230], SIRT-2 PDB:1J8F [6], SIRT-3 PDB:5D7N [309], SIRT-5 PDB:2B4Y [314], SIRT-6 PDB:3K35 [291] and SIRT-7 PDB:5IQZ [297]). Three hits were selected on the basis of their drug-like and ADME profile: in particular, **Sulforaphane** showed therapeutic potential against SIRT-1 and -5, **Kaempferol** on SIRT-3 and **Apigenin** against SIRT-6. In vitro studies confirmed the hypothesized activities.

### 3.9. Parasitic Sirtuins

In addition to human isoforms, parasitic sirtuins represent an important target to treat various protozoa-related diseases [315,316,317,318,319,320]. This is due to the importance of sirtuins in different physiological processes of the parasite [321], but an important warning is represented by selectivity issues toward the host’s isoforms. Here, we report VS studies targeting *Trypanosoma cruzi*, *Schistosoma mansoni* and Leishmania sirtuins.

In **2012**, Soares et al. performed a docking-based VS of a series of nicotine analogues toward *Trypanosoma cruzi* sirtuin TcSir2rp3 [281]. An HM of the target was used as a receptor model for the VS. The top hit of the screening resulted to be nicotinamide itself. In vitro tests confirmed the antitrypanosomal activity of this compound. **A few years** later, Sacconnay et al. [282] performed an SBVS of 50 natural trypanocydal compounds against both of the two sirtuins of *Trypanosoma cruzi* (TcSIr2rp1 and TcSir2rp3). Two homology models corresponding to the two isoforms were employed as a docking template for the VS. The aim of the study was to evaluate if the anti-parasite action of the screened compounds could be exerted via Sirtuin inhibition. The score of four molecules (an anacardic acid derivative, aculeatin D, 16-acetoxy-11-hydroxyoctadeca-17-ene-12,14-diynylethanoate and vismione D) exceeded the one of AGK2 and thiobarbiturate 6 (two potent and selective inhibitors of sirtuins, respectively). For these compounds, a sirtuin-mediated mechanism was hypothesized: nevertheless, experimental validation is required. VS techniques were **further applied** to develop new inhibitors of *Schistosoma mansoni* Sirtuin 2, a protein involved in the reproductive functions of this parasite [283]. An HM was used to screen a zinc [226]-derived library. As species-selective compounds are desired, an analogous screening was performed on the human SIRT-2. Four leads were selected by comparing the two screening results, and by score comparison with respect to the AGK2-hSIRT-2 complex. In **2008**, Kadam et al. performed a combined LB-SB VS targeting Leishmania sirtuin [280]. In particular, the fingerprint of nicotinamide was used as a query to screen the NCI database [322]. The retrieved molecules were docked in the hSir2 crystal structure [6] and in a previously reported [323] HM of LmSir2. The results were grouped according to their putative selectivity, and members of each group were submitted to experimental validation. Despite the fact that a truly potent and selective lead was not individuated, some of the tested compounds showed potential for further optimization.

## 4. Conclusions

Sirtuins are involved in a wide range of different processes, ranging from transcription to metabolism to genome stability. Their dysregulation is thought to be related to the pathogenesis and/or progression of different diseases, such as cancer, neurodegenerative disorders and T2D. In this context, molecules targeting selectively different SIRTs are considered as promising compounds for the search for new drugs. Up to now, the discovery of novel SIRT modulators was mainly addressed by applying different rational methods and VS strategies, in the attempt of identifying hit compounds for the further hit-to-lead optimization process. This approach allowed for the discovery of different chemo-types, worthy of further investigation and development.

Most of the VS herein presented regarding the search for SIRT-1 modulators relied on structure-based studies, a number of X-ray crystallographic data being available about SIRT-1 in the presence of activator/inhibitors. Indeed, six and three of the reported studies, out of 14 reported analyses (see Table 3), have been performed by SBVS and combined LB-SB VS strategies, respectively. In most of them, inhibitors as well as activators have been successfully identified. Conversely, SIRT-2 modulators have been discovered mainly thanks to combined LB-SB VS rather than SBVS (5 and 7 studies out of the 12 reported), highlighting on the other hand the predominant effective role played by the high number of structural information on SIRT-2-inhibitor complexes. During the last years, docking programs have been successfully embedded in automated workflows for ultra-large compound library screening, confirming the first steps of the VS search as viable. However, the selection of promising virtual hits (usually less than 100 compounds) from many high-scoring compounds in the library remains a challenge, since different selection protocols usually lead to different results.

Despite the consistent number of similar data regarding SIRT-6, few series of novel derivatives have been so far identified, the search for deepening studies including molecular dynamic simulations being an urgent need to properly manage this issue as well as the selectivity profile of any new putative SIRT-6 modulator. Lastly, performing in silico the prediction of the pharmacokinetic and toxicity (ADMET) properties of compounds should be performed in parallel, starting from the first steps of the drug design process and VS studies: compound library collection and the following hit-to-lead analysis would be efficiently oriented, as more drug-like compounds, prior to chemical synthesis.

## Figures and Tables

**Table 1:** Enzymatic activities and cellular localizations of the 7 sirtuins.

Sirtuin	Enzymatic Activity	Cellular Localization
**SIRT-1**	Deacetylase [7]	Nucleus, Cytoplasm [13,14]
**SIRT-2**	Deacetylase, Deacylase [13,15,16]	Nucleus, Cytoplasm [13,15]
**SIRT-3**	Deacetylase [17,18]	Mitochondria [17,18]
**SIRT-4**	Mono-ADP-ribosyltransferase, Lipoamidase [19,20]	Mitochondria [19]
**SIRT-5**	Deacylase, Desuccinylase, Demalonylase [21,22]	Mitochondria [23]
**SIRT-6**	Deacetylase, Deacylase, Mono-ADP-ribosyl transferase [24,25,26,27]	Nucleus [13,14]
**SIRT-7**	Deacetylase [19]	Nucleus (nucleolus) [13,14]

**Table 2:** Overview of the pharmacological modulation of sirtuins with a therapeutic effect in cancer, neurodegenerative diseases (Alzheimer’s disease—AD, Huntington’s disease—HD, Parkinson’s disease—PD, Amyotrophic Lateral Sclerosis—ALS, Multiple Sclerosis—MS) and type 2 diabetes (T2D).

Sirtuin	Cancer	Neurodegenerative Diseases (AD, HD, PD, ALS, MS)	Type 2 Diabetes (T2D)
**SIRT-1**	Activation or inhibition (depending on cancer type)	AD: activation HD: inhibition PD: activation ALS: inhibition MS: inhibition	Activation
**SIRT-2**	Inhibition	AD: inhibition HD: inhibition PD: inhibition ALS: - MS: -	Inhibition
**SIRT-3**	Activation or inhibition (depending on cancer type)	AD: activation HD: - PD: activation ALS: - MS: activation	Activation
**SIRT-4**	-	-	-
**SIRT-5**	Activation or inhibition (depending on cancer type)	-	-
**SIRT-6**	Activation or inhibition (depending on cancer type)	AD: - HD: - PD: inhibition ALS: - MS: inhibition	Inhibition
**SIRT-7**	Inhibition (depending on cancer type)	-	-
